# A weak coupling mechanism for the early steps of the recovery stroke of myosin VI: A free energy simulation and string method analysis

**DOI:** 10.1371/journal.pcbi.1012005

**Published:** 2024-04-25

**Authors:** Florian E. C. Blanc, Anne Houdusse, Marco Cecchini

**Affiliations:** 1 Institut de Chimie de Strasbourg, UMR7177, CNRS, Université de Strasbourg, Strasbourg, France; 2 Structural Motility, Institut Curie, CNRS, UMR144, PSL Research University, Paris, France; Babes-Bolyai University: Universitatea Babes-Bolyai, ROMANIA

## Abstract

Myosin motors use the energy of ATP to produce force and directed movement on actin by a swing of the lever-arm. ATP is hydrolysed during the off-actin re-priming transition termed recovery stroke. To provide an understanding of chemo-mechanical transduction by myosin, it is critical to determine how the reverse swing of the lever-arm and ATP hydrolysis are coupled. Previous studies concluded that the recovery stroke of myosin II is initiated by closure of the Switch II loop in the nucleotide-binding site. Recently, we proposed that the recovery stroke of myosin VI starts with the spontaneous re-priming of the converter domain to a putative pre-transition state (PTS) intermediate that precedes Switch II closing and ATPase activation. Here, we investigate the transition from the pre-recovery, post-rigor (PR) state to PTS in myosin VI using geometric free energy simulations and the string method. First, our calculations rediscover the PTS state agnostically and show that it is accessible from PR via a low free energy transition path. Second, separate path calculations using the string method illuminate the mechanism of the PR to PTS transition with atomic resolution. In this mechanism, the initiating event is a large movement of the converter/lever-arm region that triggers rearrangements in the Relay-SH1 region and the formation of the kink in the Relay helix with no coupling to the active site. Analysis of the free-energy barriers along the path suggests that the converter-initiated mechanism is much faster than the one initiated by Switch II closure, which supports the biological relevance of PTS as a major on-pathway intermediate of the recovery stroke in myosin VI. Our analysis suggests that lever-arm re-priming and ATP hydrolysis are only weakly coupled, so that the myosin recovery stroke is initiated by thermal fluctuations and stabilised by nucleotide consumption via a ratchet-like mechanism.

## Author summary

Myosin is an ATP-powered motor protein that is crucial for cellular functions like muscle contraction, cell division, and the transport of molecular cargos. Understanding how myosin transforms chemical energy from ATP hydrolysis into mechanical work is a central open question in structural bioenergetics, which could guide the design of next-generation synthetic nanomachines. In myosin, ATP hydrolysis is coupled to the re-priming of the lever-arm during the recovery stroke transition. Using advanced molecular dynamics simulations, we described the sequence of events and the energetics at an early stage of the recovery stroke of myosin VI with an unprecedented level of detail. The results support a mechanism in which the re-priming of the mechanical amplifier region of the protein is almost entirely driven by thermal fluctuations and stabilized by ATP hydrolysis. This weakly-coupled mechanism suggests that myosin can “rectify” thermal fluctuations to work efficiently in an isothermal environment dominated by stochastic fluctuations like the cell.

## Introduction

**Fig 1:**
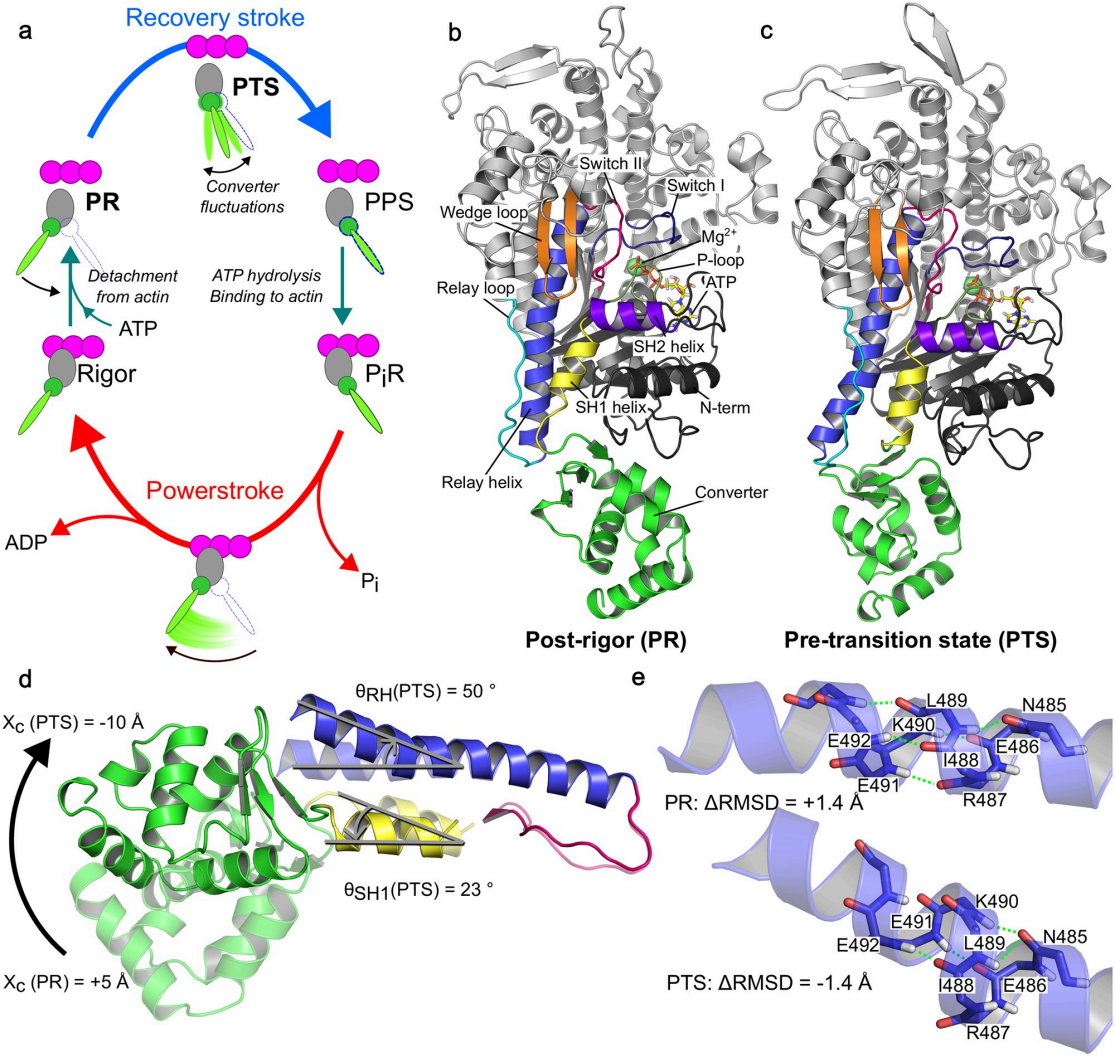
Overview of the PR → PTS transition. (a) Simplified Myosin VI functional cycle including the putative PTS. (b) Equilibrated PR structure highlighting the structural elements involved in the recovery stroke. (c) Equilibrated PTS structure, colored as in b. (d) Comparison between PR (transparent) and PTS (opaque) showing the movement of the converter, the tilting of the SH1 helix, and the kink and re-orientation of the C-terminal fragment of the Relay Helix. Typical values for the corresponding CVs are given. (e) Comparison of the backbone structure of residues 485–491 of the Relay helix before and after kink formation. Typical values of the ΔRMSD are given.

Myosins are a superfamily of actin-based motor proteins involved in cellular functions such as cargo transport, endocytosis, cell division and motility [1]. Force and directional movement are produced upon strong binding of the motor domain to F-actin, which triggers the sequential release of the ATP hydrolysis products and the forward movement of the lever-arm domain, or powerstroke [1–3], see Fig 1A. Binding of a new ATP molecule promotes detachment from F-actin and initiates the recovery stroke, *i.e.*, the transition during which the lever-arm is re-primed to its armed configuration. In absence of actin, this transition is rather fast (≈1 ms) and reversible. Interestingly, ATP is hydrolyzed at the end of the recovery stroke rather than during the powerstroke. Therefore, the mechanistic elucidation of this complex conformational transition is necessary to understand the structural basis for chemo-mechanical transduction in myosin. Myosin VI (Myo6) is the only-known minus-directed motor, and fulfills specific cellular roles [4, 5]. Myo6 harbors two unique inserts including one responsible for directionality reversal, but it otherwise shares the same design plan as other myosins [6–8].

High-resolution crystal structures of the initial state (post-rigor state, PR) and final state (pre-powerstroke state, PPS) of the recovery stroke of *Dictyostelium discoideum* Myosin II (Dd Myo2) as well as porcine Myo6 have revealed the main subdomain rearrangements, which are similar for both motors. Chief among them are: the re-priming of the converter/lever-arm; the formation of a kink halfway along the conserved Relay helix (RH); and the closure of the Switch II loop over the *γ*-phosphate of ATP [7, 9]. This latter rearrangement turns on ATPase function by stabilizing an active site geometry conducive to hydrolysis [10–13]. A key open question is how Switch II closure couples to converter re-priming. To date, most computational analyses of the PR→PPS transition mechanism have predicted that Switch II closure initiates the recovery stroke [14–25]. Among them, the popular model by Fischer and co-workers posits a strongly, or *mechanically* coupled mechanism where Switch II closure directly drives the rotation of the converter [14, 17]. In this model, structural changes occur in a progressive, concerted fashion and involve a two-step rearrangement in the RH, which sits between the active site and the converter. Based on the combination of X-ray crystallography, all-atom MD, and free-energy calculations we have recently hypothesized an alternative mechanism for the recovery stroke of Myo6 in which the rotation of the converter precedes the closure of Switch II and is uncoupled from it until the late stage of the transition [26]. Because this mechanism entails near complete converter re-priming driven purely by random fluctuations, we named it the “ratchet-like” model. This model postulates the implication of a novel structural intermediate called pre-transition state (PTS), which exhibits hallmarks of the post-recovery state, namely a (nearly) re-primed converter and a kink in the RH yet with an open Switch II; see Fig 1. And, it is based on the observation that the free energy barrier for closing Switch II over ATP in PR is as high as 12 kcal/mol. Intriguingly, such a high barrier suggests that a converter-initiated, ratchet-like mechanism could be explored faster, thus contributing to the main transition pathway. Under the ratchet-like model, the recovery stroke mechanism would comprise two major conformational transitions, the PR → PTS transition in which (most of) the converter re-priming takes place, and the PTS → PPS transition in which Switch II closes. In this scenario, converter swing is weakly, or *statistically*, coupled to Switch II closure. Whether the recovery stroke of Myo6 proceeds via a strongly-coupled, Switch II-initiated or statistically-coupled, converter-initiated mechanism remains an open question.

To explore the recovery stroke mechanism with atomic resolution, we turn to all-atom MD simulations augmented by enhanced sampling strategies. Geometric free energy calculations (including the adaptive biasing force method, or ABF) evaluate the potential of mean force (PMF) along low-dimensional collective variables (CVs) describing the transition [27–30]. The related string method in collective variables (CVSM) identifies the minimum free energy path (MFEP) in a CV-space of arbitrary dimension [31, 32], which makes it ideal to elucidate the transition mechanism between two known structures. The string method has been used to describe conformational transitions in molecular machines; see for example [33–35]. For myosin, Ovchinnikov et al. applied it to the internal conformational transition of the Myo6 converter [36, 37] and Cui et al. used it in combination with a quantum-mechanical energy function to map ATP hydrolysis pathways in Dd Myo2 [12]. To our knowledge, the string method has not been applied yet to the elucidation of conformational transitions of the full-length myosin motor domain.

Here, we report on a computational analysis of the mechanism and energetics of the transition from PR to PTS, *i.e.*, the first major step of the recovery stroke in the ratchet-like model. First, we use ABF to map a coarse-grained free energy landscape of the transition. Consistent with the ratchet-like hypothesis, we identify agnostically a state corresponding to PTS, and show that it is accessible from PR by a low free energy path, supporting its relevance as an on-pathway intermediate. Second, we perform path optimizations with the CVSM to determine a higher-resolution description of the transition. These independent calculations agree remarkably well with the ABF map and reveal a plausible mechanism by which near-complete re-priming of the mechanical amplifier region is triggered by spontaneous converter/lever-arm swing, eventually resulting in the formation of the kink in the RH while Switch II remains open. Despite some uncertainty as to the exact nature of the rate-limiting step (lever-arm movement vs formation of the kink), the free energy barrier for converter swinging (4–6 kcal mol^−1^) is consistently found to be lower than that for closing Switch II from PR (12 kcal mol^−1^), which suggests that the recovery stroke is more likely to start with a movement of the converter than closure of Switch II. Our results support the PTS state as a functional intermediate and the ratchet-like model as a plausible mechanism to understand the recovery stroke of myosin VI.

## Results

To investigate the recovery stroke of Myo6, we combine extended ABF (eABF, [38]) and the CVSM to take advantage of their respective strengths. We introduce two CVs: *X*_*c*_ (Fig 1D) to measure the position of the converter relative to the motor domain and Δ*RMSD* (Fig 1E) to describe the local conformation of the RH (*i.e.,* the formation of the kink); see Methods for a precise definition. These CVs describe two characteristic rearrangements from PR to PTS. We perform an eABF calculation over (*X*_*c*_, Δ*RMSD*) to determine the coarse-grained free energy landscape of the early recovery stroke in the ratchet-like model, starting from the PR state. Then, we carry out string optimizations in a higher-dimensional space encompassing degrees of freedom left out of the eABF calculation, to determine a high-resolution picture of the transition mechanism from PR to PTS. This strategy yields an integrated structural and energetic description of the early stage of the recovery stroke and enables consistency checks between separate sets of simulations.

### Coarse-grained free energy landscape of the PR → PTS transition

The full coverage of the configurational space, smooth convergence of the free energy gradient estimates and low estimated statistical errors suggest that the eABF calculations are converged (see S1 Text, Section 1 and S1, S2 and S3 Figs).

#### eABF discovers the PTS state without prior knowledge

**Fig 2:**
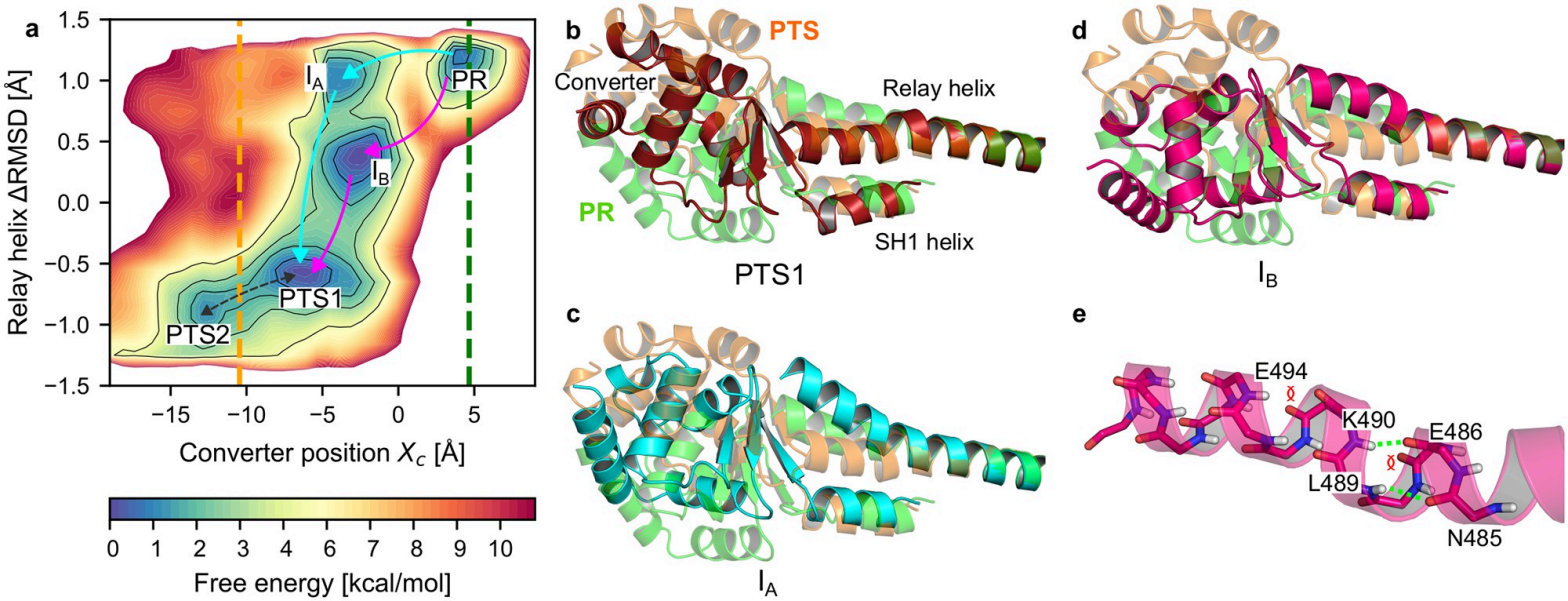
Analysis of the PR → PTS transition by eABF. (a) Free energy landscape obtained by eABF with metastable states labelled. The dotted green (respectively orange) line materializes the value of *X*_*c*_ in the PR crystal structure (respectively PTS crystal structure). The arrows indicate putative transition pathways, namely Path *A*_*ABF*_ (cyan) and Path *B*_*ABF*_ (pink). (b-d). Representative conformations (average structure of the most populated cluster of the corresponding state, see Methods and S1 Text, Section 1) of the converter and Relay and SH1 helices of the PTS1 (b, dark red), I_*A*_ (c, cyan) and I_*B*_ (d, bright pink) metastable states, compared to PR (transparent green) and PTS (transparent orange) equilibrated structures. Structures are aligned onto the N-terminal fragment of the Relay helix (residues 468–482) to better isolate changes in Relay and SH1 helix orientation and converter position. (e) Close-up on the disruption of the Relay helix in I_*B*_.

The free energy landscape computed by eABF reveals 5 local free energy minima, including the PR basin (upper right corner), see Fig 2A. Strikingly, a wide basin encompassing 2 sub-minima is found in the lower left corner of the free energy landscape, corresponding to a kinked RH and (partially) re-primed converter. Interestingly, we observe that the active site -as characterized by the *d*_1_ and *d*_*γ*_ distances, see Methods- remains open throughout eABF sampling, including in the identified metastable states (S4a Fig). Therefore, the calculations indicate that there exists a metastable Myo6 configuration with open active site, nearly re-primed converter and kinked RH. We note that if the closure of Switch II were strongly coupled to converter movement and/or RH kink formation, closed active-site conformers would have been preferentially sampled in the lower-left region of the free energy landscape, which is not the case. Additionally, this basin’s characteristics are consistent with the PTS state, as evidenced by comparison to previously reported unbiased MD simulations of Myo6 [26], see S4b Fig. We find that both sub-basins (labelled PTS1 and PTS2 in Fig 2A) correspond to converter positions and RH conformations explored in unbiased PTS simulations. Finally, representative conformers extracted from the PTS1 basin by clustering demonstrate marked structural similarity to the PTS crystal structure (Fig 2B). Taken together, these findings imply that not only a partially re-primed state with open active site exists, but also that the PTS structure is representative of this state, indicating that eABF has discovered the PTS state without prior knowledge of the PTS structure (see Methods). The finer structure of the free energy landscape suggests -also in agreement with earlier unbiased MD [26]- that the PTS state encompasses at least two distinct metastable states, PTS1 and PTS2, separated by a low, 3 kcal mol^−1^ free energy barrier, which correspond to slightly different RH conformers and average converter positions. The PTS2 converter position is closer to PPS, suggesting it is on the recovery stroke pathway. The transition from PTS1 to PTS2 entails a further 7 Å converter movement associated with a slight re-orientation of the RH. This transition corresponds to rapid fluctuations within the PTS basin, as demonstrated by their reversible sampling on the ∼ 100 ns timescale in earlier unbiased MD simulations, and agrees with our previous observation that the converter is highly dynamic in the PTS state [26].

#### Transition tube and mechanism

The free energy landscape shows that a low free energy transition tube connects PR to PTS. This indicates that the PTS state is kinetically accessible from PR and supports the hypothesis that it is an on-pathway intermediate along the recovery stroke. Two local free energy minima are identified within the transition tube, labelled I_*A*_ and I_*B*_ on Fig 2A. We use structural clustering to reveal their structural features. This analysis indicates that I_*A*_ corresponds to an intermediate along the PR→ PTS transition where the converter has moved by 7.8 Å in the re-priming direction, promoting partial tilting of SH1 and bending of the RH in response, but not yet formation of the kink (Fig 2C). In I_*B*_, the converter has moved by only 6.5 Å and its orientation is quite different from I_*A*_, and closer to PR (Fig 2D and S5a Fig). Moreover, in I_*B*_ the RH backbone differs both from the regular helical structure observed in PR and the kink observed in PTS (and PPS) crystal structures. Rather, the RH exhibits local unfolding and bending near residues 490 to 494 (*i.e*, ∼ 5 residues in C-ter of the canonical kink), see Fig 2E and S5b Fig.

Although the transition tube is suggestive of a sequential mechanism with I_*A*_, then I_*B*_ as on-pathway intermediates, the differences in converter position/orientation and RH structure are also consistent with I_*A*_ and I_*B*_ belonging to two different pathways (Paths *A*_*ABF*_ and *B*_*ABF*_, respectively in cyan and pink on Fig 2). In Path *A*_*ABF*_, the initiating event is a ≈8 Å movement of the converter which crosses the highest free energy barrier along this path (6 ± 1 kcal mol^−1^, see S3 Fig for statistical error estimation), suggesting it may represent the rate-limiting step. The second step to reach PTS1 is the formation of the kink in the RH, which contributes an additional ≈2 Å of converter swing. In Path *B*_*ABF*_, the first step is a 6.5 Å movement of the converter associated with local disruption of the RH backbone, and the second step corresponds to the formation of the “canonical” kink in the RH along with a further 3.5 Å converter swing to PTS1. Despite their differences, both paths predict an early converter movement as the first step of the recovery stroke in Myo6. Notably, our previous unbiased simulations of the PR state did capture spontaneous converter uncoupling without RH kinking [26], which may indicate that Path *A*_*ABF*_ is more likely to represent the dominant mechanism to reach PTS (S4 Fig, simulation PR+ATP (3)).

The analysis of the free energy landscape allows us to: 1) validate the relevance of PTS as an accessible metastable state from PR and 2) draw a first mechanistic picture of this transition. However, it does not reveal how other important structural rearrangements, such as changes in the SH1 helix, are coupled to the transition. We now address this with the string method in collective variables.

### String method analysis of the PR → PTS transition

First, we performed preliminary string optimizations in (*X*_*c*_, Δ*RMSD*), which showed that the minimum free energy path (MFEP) for the PR → PTS transition lies within the transition tube predicted by eABF (S7 and S8 Figs and S1 Text, Section 2). This independent agreement confirms that the general features of the transition predicted by eABF are robust. Then, we set out to describe the transition mechanism with higher resolution. To this end, we used the string method with 12 collective variables giving a more detailed insight into the rearrangements involved in the PR → PTS transition (Table 1; we note that Δ*RMSD* was not used as a supporting CV for 12D CVSM calculations because the 4 kink distances serve the same purpose, but with better descriptive power).

**Table 1:** List of collective variables defined in the study of the myosin VI recovery stroke. All distances, orthogonal projections and RMSD are in Å. All angles are in deg.

Collective Variable	Rearrangement	Biased in	Force constant (kcal/mol/U^2^)
Orthogonal projection *X*_*c*_	Converter swing	eABF, string (2d, 12d, 20d)	10
Orthogonal projection *Y*_*c*_	Converter swing	string (12d, 20d)	10
Orthogonal projection *Z*_*c*_	Converter swing	string (12d, 20d)	10
Distance *i*_1_ (65O:707OG)	Converter/main body interaction	string (20d)	20
Distance *i*_2_ (66CE:F763 phenyl ring)	Converter/main body interaction	string (20d)	20
Distance *i*_3_ (65O:702NE2)	Converter/main body interaction	string (20d)	20
Distance *i*_4_ (66O:702NE2)	Converter/main body interaction	string (20d)	20
Distance *i*_5_ (64O:761N)	Converter/main body interaction	string (20d)	20
Orientation angle *θ*_*RH*_	Orientation of the Relay helix	string (12d, 20d)	5
Orientation angle *θ*_*SH*1_	Orientation of the SH1 helix	string (12d, 20d)	5
Distance *d*_*R*/*SH*1_ (469–482CA:693–703CA)	Relay/SH1 seclusion	string (12d, 20d)	20
Orthogonal projection *Z*_*SH*1_	SH1 piston motion	None	N.A.
Relay helix Δ*RMSD*	Relay helix kink	eABF, string (2d)	125
Distance *k*_1_ (486O:490N)	Relay helix kink	string (12d, 20d)	50
Distance *k*_2_ (485O:489N)	Relay helix kink	string (12d, 20d)	50
Distance *k*_3_ (485O:490N)	Relay helix kink	string (12d, 20d)	50
Distance *k*_4_ (486O:491N)	Relay helix kink	string (12d, 20d)	50
Distance *k*_5_ (490O:494N)	Relay helix secondary kink	string (20d)	50
Distance *k*_6_ (491O:495N)	Relay helix secondary kink	string (20d)	50
Distance *k*_7_ (492O:496N)	Relay helix secondary kink	string (20d)	50
Dihedral *χ*_11_ (L489 N:CA:CB:CG)	Hydrophobic switch	string (12d, 20d)	0.01
Dihedral *χ*_12_ (L700 N:CA:CB:CG)	Hydrophobic switch	string (12d, 20d)	0.01
Distance *d*_1_ (R205CZ:E461CD)	Critical salt-bridge	None	N.A.
Distance *d*_*γ*_ (G459N:ATPO1G)	Switch II-ATP hydrogen bond	None	N.A.

The starting (or guess) path for string optimizations was obtained by “uplifting” and regularizing the 2-dimensional string optimized over the eABF free energy landscape to the 12-dimensional space (S1 Text, Section 2). Two separate string optimizations, with slightly different parameters, were performed until convergence (S9 Fig, S1 Text, Section 2). Visual analysis of the structures along averaged strings over the last 50 iterations shows that the two calculations converged towards a similar sequence of events, which represents the most likely pathway for the PR → PTS transition of Myo6, and which we call Path A (S11 Fig). Backprojection of the two converged Path A strings (Strings A1 and A2) onto the (*X*_*c*_, Δ*RMSD*) plane reveals that they both lie within the eABF-predicted transition tube. Interestingly, the two converged strings visit I_*A*_, but not I_*B*_, see S10 Fig. To obtain an energetic view of the transition mechanism, we then performed an umbrella sampling calculation along string A1 with a finer discretization (from 32 to 128 equally-spaced images) and computed the free energy profile along the string, and 2D free energy maps along pairs of observables (see Methods and S1 Text, Section 3). From the analysis of string A1, we now describe the sequence of events, structural couplings, and energetics governing the PR → PTS transition.

#### Overview of the mechanism, free energy profile and comparison with eABF

**Fig 3:**
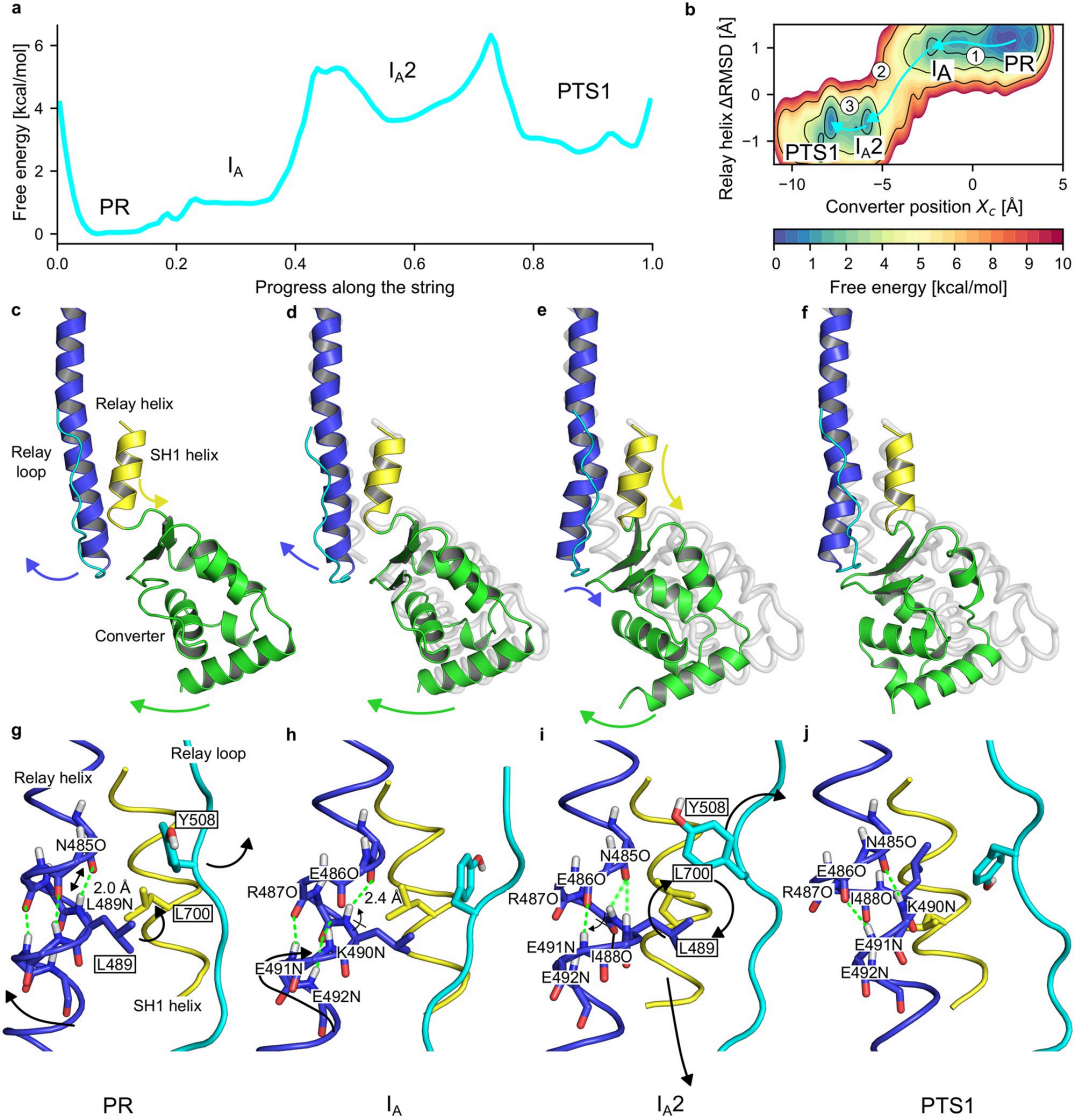
Free energy profiles and mechanism of the PR → PTS transition. (a) PMF along the path obtained by Umbrella Integration. (b) 2D PMF along (*X*_*c*_, Δ*RMSD*) obtained by MBAR. Arrows indicate transition steps between metastable states. (c-f) Sequence of metastable states in the PR → PTS transition from string calculations. Arrows indicate the movements of structural elements. For easier comparison, the PR structure is shown in transparent grey. (g-j) Mechanism of formation of the kink in the RH. Arrows indicate backbone and side chain movements. H-bonds are shown as green dotted lines, shading indicating weak binding. (c,g) From PR, in response to the first converter movement, the RH C-ter shifts and rotates, which extends the N485O:L489N H-bond and brings L489 closer to L700. (d,h) In I_*A*_, the second converter movement translates into corkscrew movement of the RH, shifting K490N and E491N by a quarter of helical turn, but complete rotation of RH is prevented by the L489/L700 contact. Instead, the I488-L489 peptide bond flips, which breaks the I488O:E492N H-bond. This deprives R487O and I488O from H-bond partners, and weakens the N485O:L489N H-bond because of unfavorable angle. In counterpart, non-canonical H-bonds E486O:E491N and N485O:K490N form. (e,i) In I_*A*_2, structural tension is relieved by movements of the SH1 helix and the Relay loop. This frees sterical constraints on the L489 and L700 side chains, which exchange their positions while remaining in contact. I488-L489 flips back, allowing the I488O:E492N H-bond to reform. (f,j) In PTS1, the hydrophobic switch is rearranged, stabilizing the kink in the RH. R487O is free of H-bond partner. Frames in panels c-j are structures closest to umbrella sampling window-averages and are available in Zenodo.

The free energy profile along string A1 computed by Umbrella Integration [39] shows 4 metastable states (Fig 3A), indicating a 3-stage mechanism. Projection of the umbrella sampling simulations along string A1 onto the (*X*_*c*_, Δ*RMSD*) with MBAR [40, 41] reveals a free energy landscape very similar to the eABF one, but with a number of remarkable differences (Fig 3B). Free energy minima corresponding to states PR, I_*A*_ and PTS1 are identified. PTS2 is not detected because the string does not extend to this basin. I_*B*_ is not seen despite its position in configurational space being clearly sampled. This confirms the proposal that I_*A*_ and I_*B*_ do not belong to the same transition pathway. Comparison of I_*A*_ representative structures sampled independently in the eABF and string calculations reveals striking similarity (S6 Fig), which confirms that the same metastable intermediate is picked up by both calculations and justifies using the same denomination indifferently. We conclude that Path *A*_*ABF*_ and Path A are virtually identical, and we can now detail the transition mechanism.

**Step 1: PR → I_*A*_: Converter rotation re-orients Relay and SH1 helices**. Starting from the PR state, the first step is a thermally-activated (stochastic) ≈8 Å movement of the converter leading the motor domain into the I_*A*_ intermediate (Fig 3C and 3D and S11 Fig). This is consistent with the eABF prediction, but the free energy barriers differ, with umbrella sampling indicating a ≈1 kcal mol^−1^ barrier (Fig 3A), much smaller than the 6 kcal mol^−1^ eABF barrier (Fig 2). The converter displacement reduces the total number of contacts it makes with the N-terminal subdomain (S12a, S12b and S12e Fig) which increases the converter’s positional freedom relative to PR, because it is attached to the main body only through the flexible Relay and SH1 helices. The most energetically costly event in the PR → I_*A*_ step seems to be the disruption of the converter/N-terminal contacts. Since these are mostly non-specific hydrophobic interactions, the corresponding free energy barrier is rather small and can probably be crossed reversibly on relatively short timescales. This is consistent with spontaneous ∼ 100 ns converter uncoupling in unbiased MD of PR we previously reported [26]. Although I_*A*_ is in fast interconversion with PR, it represents an important intermediate because converter uncoupling from the main body is a pre-requisite to facilitate its swing.

Both the RH C-ter moiety (residues 489 to 499) and the SH1 helix re-orient concurrently with the converter movement (Fig 3C and 3D and S11 Fig). The C-ter moiety of the RH is strongly bound to the converter through specific contacts, and its N-ter moiety is anchored within the main body. As a result, the RH bends following the movement of the converter. RH bending entails only limited backbone perturbation, with a transient extension of the N485O:L489N hydrogen bond close to the bending point (Fig 3G and 3H and S11 Fig). This enables a “corkscrew”-like rotation of the RH C-ter moiety around the helical axis, which brings the L489 side chain in contact with L700 on the SH1 helix, resulting in tilting of the SH1 helix. This steric clash constitutes a first blocking point to the free movement of the converter, which is later relieved through extensive conformational changes.

**Step 2: I_*A*_ → I_*A*_2: Converter movement drives formation of the kink**. At the beginning of Step 2, a second movement of the converter is translated into further angular re-orientation of RH and SH1 (Fig 3D and 3E and S11 Fig). However, unlike Step 1, the RH backbone can no longer accommodate the strain only through bending, because this movement is blocked by the clash of L489/L700 side chains. Therefore, the “corkscrew”-like motion of the RH backbone is coupled with dramatic alterations to the H-bonding pattern, namely the formation of the E486O:E491N and the disruption of the E486O:K490N, R487O:E491N, I488O:E492N H-bonds (Fig 3H and 3I). N485O:L489N, which was extended by about 0.5 Å in I_*A*_, returns to perfect geometry. And, atoms N485O and K490N are brought closer but not yet quite enough to form a H-bond. The cost of breaking these backbone H-bonds likely explains why this step would be rate-limiting for the PR → PTS transition, as suggested by the free energy profile (highest free energy barrier, ≈4 kcal mol^−1^, Fig 3A). Upon reaching I_*A*_2, the RH is clearly kinked, even though the H-bonding pattern differs slightly from what is observed in PTS. Notably, I_*A*_2 features outward-pointing, unbound R487O and I488O backbone atoms, which may explain why I_*A*_2 is rather high in free energy (Fig 3A and 3I), even if the formation of at least one non-canonical H-bond in the RH backbone (E486O:E491N) makes this state metastable.

The analysis of the I_*A*_ → I_*A*_2 step indicates that the main driving force for the formation of the RH kink is the movement of the converter. It is plausible that capturing a large enough converter movement to drive the costly kink formation would require first putting the converter in an uncoupled state. There, the converter can efficiently harness fluctuations from the bath and amplify them into functional rearrangements within the motor domain. This illustrates why it is important to first visit I_*A*_ as an easily accessible uncoupled state.

**Step 3: I_*A*_2 → PTS1: Seclusion of the SH1 helix and rearrangement of the hydrophobic switch**. The tight packing of the L489/L700 side chains is preserved throughout the transition to I_*A*_2, including upon formation of the kink. This builds up structural frustration at the Relay/SH1 interface. During the I_*A*_2 → PTS1 transition, this frustration is relieved through two consecutive rearrangements. First, the SH1 helix moves by ≈1 Å parallel to the RH in a “piston-like” motion, and re-orients upward by ≈5 deg (Fig 3E and 3F and S11 Fig). This creates enough space to allow packing L489 against the SH1 helix. Second, a movement of the Relay loop relieves the sterical hindrance from the Y508 side chain (Fig 3I and 3J). This allows the L489 and L700 side chains to eventually exchange their positions (Fig 3I and 3J). In the process, the I488-L489 peptide bond rotates, allowing I488O to re-form a H-bond with E492N while the N485O:L489N hydrogen bond breaks. Fischer et al. and Baumketner independently proposed that a similar transition for the Dd Myo2-homologous side chains (Dd Myo2 F487, F506 and I687) stabilizes the kink [14, 24]. By analogy to the “aromatic switch” of Fischer et al. [14], we call this triplet of side chains the “hydrophobic switch” for Myo6. The sequence of events captured by our calculations for L489 and L700 is reminiscent of the mechanism proposed by these investigators, but not entirely consistent with it (see Discussion). Because the Relay loop is very flexible, its movement is likely to be a stochastic, low-barrier event weakly coupled to the rest of the transition. Thus, the movement of the SH1 helix, driven by the push of L489, likely accounts for most of the ≈3 kcal mol^−1^ barrier (Fig 3).

Overall, the A1 string calculation captures a converter swing of 14.7 Å, representing 61.6% of the total swing from PR to PPS (see S1 Table for details). This is very close to the PTS crystal structure (15.3 Å or 64.2% of the total swing).

#### No coupling to the active site is detected

**Fig 4:**
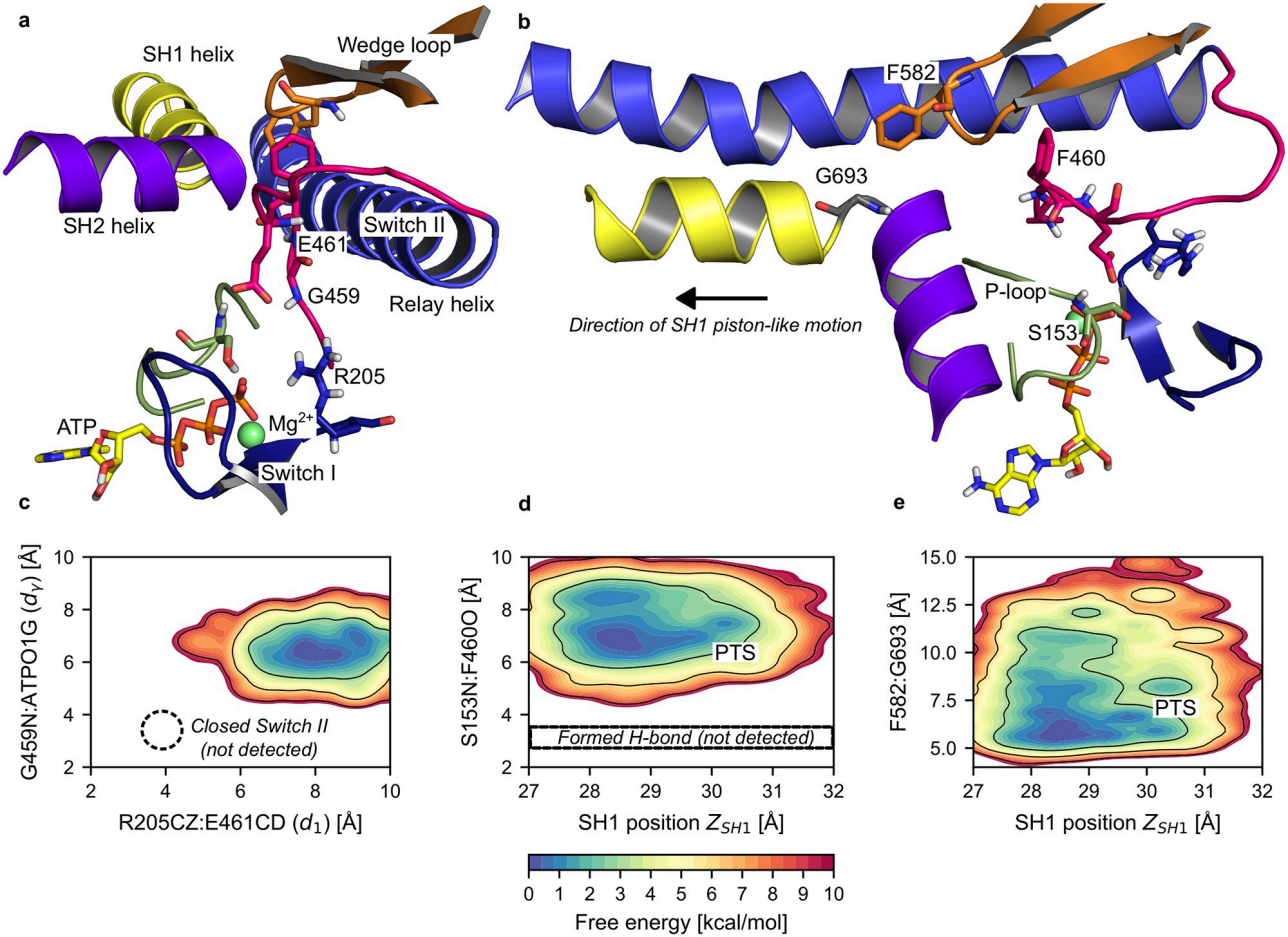
The active site remains open throughout the PR→PTS transition. (a) Close-up on the active site for a representative PTS configuration from umbrella sampling (Typical frame for image 116, see Methods). The critical salt-bridge (R205CZ-E461CD, distance *d*_1_) and the Switch II-ATP hydrogen bond (G459N:ATPO1G, distance *d*_*γ*_) are clearly not formed, corresponding to an ATPase-incompetent active site. (b) Same configuration seen from a different angle, highlighting the SH1-SH2 junction, the wedge-loop and the P-loop. Although F582 is close to G693 at the SH1-SH2 junction, the P-loop-Switch II hydrogen bond (S153N:F460O) is clearly not formed. (c) 2D PMF over *d*_1_ and *d*_*γ*_ computed along the transition. The closed Switch II state is not detected. (d) 2D PMF over the piston-like movement of the SH1 helix (*Z*_*SH*1_) and the P-loop-Switch II hydrogen bond distance (S153N:F460O) computed along the transition. The formed hydrogen bond is not detected. (e) 2D PMF over the piston-like movement of the SH1 helix (*Z*_*SH*1_) and the distance between the wedge loop and the SH1-SH2 junction (F582-G693 distance). The completion of the piston-like movement of SH1 favors closer approach between the wedge loop and the SH1-SH2 junction.

We searched for a structural response of the active site to the rearrangements of the PR → PTS transition. First, we computed the free energy map along the distances defining the catalytically-competent configuration from umbrella sampling (Fig 4A and 4C), which shows that the position of Switch II is nearly unaffected by the transition, like our observations from eABF. Then, we looked into a possible communication pathway between SH1 and the active site proposed by Fischer et al. for Dd Myo2 [17]. These authors proposed that the “piston-like” motion of the SH1 helix translates into the formation of a hydrogen bond between P-loop and Switch II, which may contribute to ATPase activation (see also Discussion). To assess whether a similar mechanism is at play during the PR → PTS transition, we computed the free energy map along the SH1 helix longitudinal projection *Z*_*SH*1_ (accounting for the piston-like motion) versus the S153N:F460O hydrogen bond distance between P-loop and Switch II, see Fig 4B and 4D. Although the free energy map suggests that the piston movement brings S153 and F460 slightly closer, this does not result in the formation of a hydrogen bond between these two residues. The contact between the wedge loop and the SH1-SH2 junction was proposed as the key element mediating structural communication between Switch II and the SH1 helix by Fischer et al. [17]. In our simulations, F582 on the wedge loop is brought somewhat closer to the SH1-SH2 junction as the transition proceeds (Fig 4E), but this is not sufficient to trigger the global movement of the wedge loop which would drive the formation of the P-loop / Switch II hydrogen bond. We conclude that the near-complete rearrangement of the mechanical amplifier element (Relay/SH1/Converter region), which is required to re-prime the lever-arm, proceeds without detectable effect on the active site.

#### Alternate mechanism for the PR → PTS transition

To assess the robustness of our findings to the starting point of string optimizations, we carried out a second set of string calculations where the guess path was obtained as the straight path joining PR to PTS in 12D CV-space (S1 Text, Section 2). This resulted in the so-called Path B, which is also consistent with the eABF transition tube, but does not overlap with Path A. Instead, Path B admits I_*B*_ as an intermediate and is virtually identical to Path *B*_*ABF*_, *i.e.,* a mechanism in which the RH transiently develops a secondary kink (S1 Text, Section 4, and S14 Fig). Because of this dramatic perturbation to the RH secondary structure, we expect path B to face a much larger energy penalty than path A. We therefore make the assumption that the PR → PTS transition flux is dominated by path A, which we have focussed on. This assumption is supported by the transient converter uncoupling without RH kinking observed in our earlier unbiased simulations of PR [26]. We stress that path B, like path A, describes a converter-initiated mechanism for the early recovery stroke that is weakly coupled to myosin’s active site. Moreover, if path B were dominant, its highest free energy barrier to PTS would still be lower than 4–6 kcal mol^−1^. Therefore, the most important conclusions of our study (see Discussion) do not depend on whether path A or B is more relevant. Umbrella sampling along path B, which we did not perform here, could help discriminate between the two paths. In addition, path B uniquely predicts that E494, L495 and Y496 are involved in the initial converter rotation through formation of the secondary kink. Experimental mutational perturbation of these residues might slow down the PR → PTS transition (and possibly, the whole recovery stroke) if path B were dominant.

## Discussion

Molecular motors like myosin convert chemical energy from ATP hydrolysis into mechanical work following established thermodynamic principles [42]. However, how these principles are implemented at the molecular level remains unclear. We have tackled this question using all-atom MD simulations and free energy calculations. In particular, we focused on the early stage of the recovery stroke transition, which couples the re-priming of the myosin lever-arm to ATP hydrolysis. Our results shed light on a realistic chemo-mechanical transduction mechanism, and illuminate putative structural intermediates visited during this off-actin transition, which may be targeted by allosteric modulatory ligands [43, 44].

Using advanced free energy simulations started from the X-ray structure of the post-rigor (PR) state of myosin VI, we illuminated a low-energy transition pathway connecting PR to PTS, a putative intermediate previously characterized by X-ray crystallography, with an unprecedented level of detail. Consistent with previous observations [26], our calculations suggest that the recovery stroke of myosin VI is initiated by a movement of the converter promoted by thermal fluctuations accompanied by kinking of the RH, which corresponds to the highest energy barrier, tilting of the SH1 helix, and stabilization via a switch of hydrophobic interactions between residues at the Relay/SH1 interface. Remarkably, these calculations not only “re-discover” PTS agnostically but also show that the same sequence of events could be obtained using two different free energy simulation methods, which strengthens the relevance of these findings. Most importantly, it was found that re-priming of the converter subdomain, *i.e.,* a key element of the mechanical amplifier region of the motor protein, involves a free-energy barrier ≤6 kcal mol^−1^ with an open Switch II, whereas the free energy barrier for closing Switch II over the nucleotide-binding site in PR is 12 kcal mol^−1^, as estimated in a previous study by us using a comparable approach [26]. Assuming similar pre-exponential factors, these results suggest that the converter-initiated mechanism would be ≈23 000 times faster than any Switch II-initiated scenario, indicating that converter re-priming likely precedes closure of Switch II. These observations suggest that lever-arm re-priming and ATP hydrolysis are only weakly coupled, so that the recovery stroke transition can be initiated by thermal fluctuations and driven via modulation of kinetic barriers. The latter supports the conclusion that the recovery stroke in Myo6 is plausibly mediated by a “ratchet-like” mechanism. Compared to the Switch II-initiated mechanisms, a ratchet-like mechanism would increase the motor efficiency by ensuring that ATP hydrolysis takes place only once the mechanical amplifier region is re-primed avoiding futile ATP consumption [45], and by allowing the motor to effectively harness and “rectify” ubiquitous Brownian fluctuations.

Our mechanistic proposal contrasts with previous models of the recovery stroke, most of which predicting Switch II closure as the initiating event. The mechanism proposed by Fischer and co-workers in Dd Myo2 consists of two phases [14, 17]. In phase I, a movement of the converter is coupled to Switch II closure via a rigid-body ”seesaw” movement of the RH. In phase II, an additional movement of the converter is coupled to SH1 helix tilting, RH kink formation, and the establishment of Switch II/P-loop interactions. This scenario is plausible and consistent with Dd Myo2 mutants, but it derives from zero-temperature calculations, which are inherently biased towards strong coupling. Elber and West calculated a zero-temperature transition path similar to Fischer’s model and used finite-temperature MD to evaluate the transition rate along this path [22]. Although the predicted rate is consistent with experimental estimates, these simulations are likely too short (a few ps) to appreciably deviate from the zero-temperature transition path. Woo and Harris explored the recovery stroke of Scallop myosin II by umbrella sampling. These calculations suggested weak coupling between Switch II closure and converter rotation, but it is unclear whether the short simulation trajectories (<6 ns) could faithfully reveal the sequence of events and the energetics [18, 19]. Using Targeted MD (TMD) simulations from the PR structure of Dd Myo2, Cui and co-workers predicted an early movement of the converter followed by RH kinking and Switch II closure [21], which is reminiscent of the mechanism proposed here. However, partial Switch II closure (critical salt-bridge formation) was observed at the beginning of the transition consistent with an early Switch II closure. Baumketner and Nemeslov came to the same conclusion using high-temperature simulations of the PR state in implicit solvent [23]. Later, using enhanced sampling Baumketner convincingly showed that structural rearrangements within the mechanical amplifier region are statistically coupled, but its fragmental model lacking Switch II prevented him from exploring the consequences at the nucleotide binding site [24, 25]. In brief, both methodological and sampling limitations (with respect to current state-of-art) might explain why earlier studies did not detect PTS. In addition, the usage of different myosin isoforms might also contribute to diverging outcomes.

**Fig 5:**
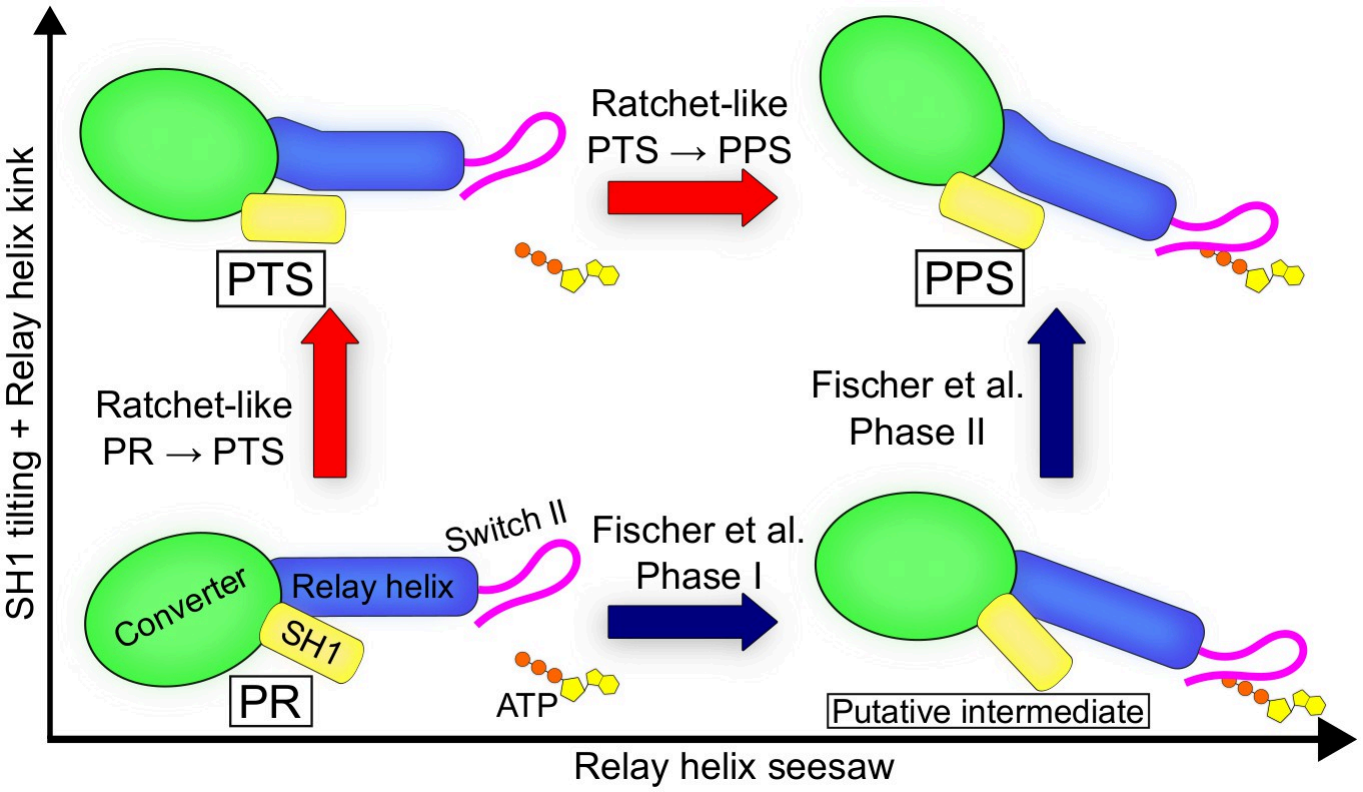
Unified mechanistic framework for the myosin recovery stroke.

Our ratchet-like model shares features with the more broadly accepted Switch II-initiated mechanisms by Fischer and coworkers, and by Baumketner, albeit with important differences. First, in the Switch II-initiated scenario, the seesaw motion of the Relay helix precedes kinking, as the latter involves threading the bulky Dd Myo2:F487 between the RH backbone and the Relay loop, which would be sterically prevented until the RH seesaw motion opens the way. By contrast, we find here that a movement of the Relay loop is sufficient to enable threading of the homologous L489 in Myo6 before the RH seesaw motion, thus resulting in an opposite order of events. Second, in the Switch II-initiated scenario it was found that SH1 tilting is coupled to the formation of a H-bond between Switch II and P-loop at the nucleotide-binding site (Dd Myo2:S183N-F458O) via the wedge loop. Yet, we do not observe the formation of the corresponding H-bond in Myo6 (Myo6:S153N-F460O) and the PTS crystal structure does not exhibit it either, suggesting that this key interaction forms during the PTS → PPS transition instead. Third, in the Switch II-initiated scenario, the tilting of the SH1 helix drives the converter rotation, whereas we come to the opposite conclusion. Fourth, in the converter-initiated scenario, the seesaw motion of the Relay helix (coupled to an additional converter movement and Switch II closure) occurs in the PTS → PPS transition, which is not explored here and left for future studies. Based on current observations, both Switch II-initiated and converter-initiated mechanisms emerge as plausible alternative scenarios depending on whether RH kinking occurs before (converter-initiated) or after (Switch II-initiated) the seesaw motion (Fig 5). In this interpretation, the PTS state would be naturally described as the post-kink, pre-seesaw intermediate along the converter-initiated pathway. A different intermediate with a partially re-primed converter, an unkinked but ”seesawed” Relay helix, and a closed Switch II would be observed along the Switch II-initiated pathway [17], although such an intermediate has never been characterized. Because we did not evaluate the free energy profile over the full recovery stroke, the present work does not prove the ratchet-like mechanism for the recovery stroke of myosin. However, our results support this model showing that the initial step of the converter-initiated scenario is much faster than that of any Switch II-initiated mechanism, at least in myosin VI.

Whether the PTS state and the ratchet-like model for the recovery stroke could be extended to other myosins remains an open question. Dd Myo2 mutants interpreted as supporting the Switch II-initiated recovery stroke do not disprove the ratchet-like pathway [26]. The same is true for pathogenic mutations disrupting the converter-Relay interactions in Human β-cardiac myosin, which would perturb the recovery stroke regardless of its mechanism, as highlighted by recent free energy calculations in Dd Myo2 [46] and β-cardiac myosin [47]. Interestingly, very recent free energy calculations on β-cardiac myosin identified a PTS-like intermediate [44]. Detailed computational investigations of the recovery stroke mechanism in other myosin isoforms would advance on this question [48]. As a bonus, these studies would enable the characterization of cryptic binding pockets for the design of myosin’s allosteric modulators [44, 49, 50]. Last, further experimental work on the recovery stroke is compellingly needed to solve the puzzle, the design of which could be inspired by predictions from simulation. Here, for instance, we predicted and characterized two new structural intermediates I_*A*_ and I_*A*_2 along the PR → PTS transition. Intriguingly, I_*A*_ is remarkably similar to the structural intermediate of smooth muscle myosin II (SMM2) trapped off-actin by the CK-571 drug [43], which features a partially re-primed converter, an “un-kinked” Relay helix and an open Switch II. The search for putative intermediates predicted in simulations, either by structural biology or time-resolved spectroscopic studies, e.g. using fluorescent probes, represents a clear direction for future investigations [51].

## Materials and methods

### Preparation of structural models and Molecular Dynamics simulations

This work uses the equilibrated models of the Myo6 motor domain reported in our previous work [26]. Here, we summarize the preparation protocol. Structural models of the Myo6 PR (PDB ID: 2VAS, [8]) and PTS (PDB ID: 5O2L) states were prepared from the corresponding crystal structures according to the protocol reported in [26]. Briefly, missing loops were built with MODELLER [52]. Then, each structure was placed in an orthorhombic box (size 144 Å x 108 Å x 96 Å) of TIP3P water molecules supplemented with Na^+^ and Cl^−^ ions to reach a 150 mM salt concentration. The final systems contain about 140 000 atoms. Energetics were described by the CHARMM36 classical force-field [53, 54]. Each system was energy-minimized with harmonic restraints on the protein atoms, followed by a 1 ns-long heating MD simulation to 300 K, then a 2 ns-long equilibration MD at constant temperature (300 K) and pressure (1 bar) during which harmonic restraints were smoothly scaled down. In the present work, only the equilibrated PR system was used to launch new simulations. All MD simulations were run with a 2 fs timestep and constrained hydrogen-covalent bonds. ABF and string method simulations were performed with NAMD 2.13 [55], using a Langevin thermostat (friction coefficient 0.1 ps^−1^) and Berendsen barostat [56] (time constant 400 fs). Umbrella sampling simulations were performed with Gromacs 2021.5 [57], using the v-rescale thermostat [58] (time constant 100 fs) and Parrinello-Rahman barostat [59] (time constant 5 ps, compressibility 4.5 x 10^−5^ bar^−1^). In all simulations, the protein was kept parallel to the axes of the simulation box by applying a harmonic restraint of force constant 1000 kcal mol^−1^ on the *orientation* quaternion, as described in the *colvars* manual [60].

### Collective variables

Collective variables (CVs) were used to characterize, and in some cases bias, the structural dynamics of Myo6, focusing on the structural elements involved in the recovery stroke (Fig 1B–1E). The complete list of CVs is given in Table 1. All CVs were computed with the *colvars* module [60] and/or MDAnalysis [61, 62].

#### Converter

The Cartesian coordinates of the converter’s center of geometry in the frame formed by the principal axes of the motor domain are denoted as *X*_*c*_, *Y*_*c*_, *Z*_*c*_ (*X*′, *Y*′*Z*′ in [26]). They capture the movements of the converter relative to the motor domain during the recovery stroke. The reader is referred to reference [26], SI Appendix, for the full description of the converter CVs. The interactions of the converter with the main body of the motor domain are described by a set of interatomic distances *i*_*j*_ (see Table 1 for details).

#### Relay/SH1 elements

Angular descriptors of the orientation of the Relay helix C-terminal fragment *θ*_*RH*_ and the SH1 helix *θ*_*SH*1_ with respect to the PR crystal structure were defined as described in [26], SI Appendix. Following Baumketner [24, 25] we introduced the distance *d*_*R*/*SH*1_ between the centers of geometry of the CA atoms of residues 469–482 (N-terminal fragment of the Relay helix) and 693–703 (SH1 helix). To characterize the “piston”-like motion of SH1 highlighted by Fischer and co-workers, we defined *Z*_*SH*1_ as the orthogonal projection of the SH1 helix center of geometry onto the longitudinal principal axis of the motor domain (*i.e.*, the same axis as for *Z*_*c*_). The local conformation of the Relay helix backbone was described by a Δ*RMSD* CV defined as:

$$\begin{array}{c} \Delta RMSD(x)\equiv RMSD(x,PTS)-RMSD(x,PR) \end{array}$$ (1)

where the *RMSD* values are computed after optimal translation/rotation and with respect to the corresponding crystal structures. For the kink of the Relay helix, Δ*RMSD* was computed on atoms C, CA, O, N (backbone heavy atoms) of residues 485 to 493. This CV takes values ≈1.4 Å in PR (straight Relay helix) and ≈−1.4 Å in PTS/PPS (kinked Relay helix), Fig 1E. Although Δ*RMSD* is defined using the PTS crystal structure as a reference, we note that the local backbone configuration of the kinked Relay helix is virtually indistinguishable between PTS and PPS (0.32 Å RMSD). For all intents and purposes, using a CV based on the PPS crystal structure [7] would have given identical results. To analyze the backbone hydrogen-bonding pattern of the Relay helix with a higher resolution than offered by Δ*RMSD*, we introduced a set of distances *k*_*j*_ (see Table 1 for details). And, we computed dihedral angles *χ*_11_ and *χ*_12_ (see Table 1) to describe side-chain rotameric transitions associated with the formation of the Relay helix kink, as pointed out previously by Fischer et al. and independently by Baumketner [14, 24].

#### ATPase site

The opening state of the Switch II loop in the active site was characterized by distances *d*_1_ and *d*_*γ*_ as defined previously [26]. *d*_1_ describes the critical salt-bridge between Switch I (R205) and Switch II (E461). *d*_*γ*_ describes the Switch II-ATP hydrogen bond. When both interactions are formed, Switch II is closed and the motor is considered ATPase-competent.

### Extended adaptive biasing force (eABF) calculations

Two-dimensional eABF calculations were run along *X*_*c*_ × Δ*RMSD* using NAMD 2.13 with the *colvars* module. We prioritized *X*_*c*_ for the eABF calculation as it is the converter component which exhibits the largest variation from PR to PTS, with typical values of 5 Å in PR and −10 Å in PTS/PPS. Briefly, ABF records the average generalized force applied by the system onto the collective variables, then applies an exactly opposite bias to enhance the sampling by flattening the free energy landscape. The free energy profile is recovered by numerical integration of the free energy gradient estimate, which is the opposite of the collected average force. In extended ABF, an extended degree of freedom is harmonically coupled to the collective variable [38]. The generalized thermodynamic force felt by the extended degree of freedom is the harmonic coupling force, which can be easily evaluated. eABF then applies the standard ABF dynamics on the extended degree of freedom. eABF does not require collective variable component orthogonality, which allows us to use a Δ*RMSD* CV. Moreover, it favors smooth convergence of the biasing force estimate [38]. We used the corrected-*z*-averaged restraint (CZAR) estimator to deconvolve the free energy gradient estimate [38] and the *abf_integrate* tool to integrate it. eABF calculations were run with SHAKE active and a 2 fs timestep. The *X*_*c*_ collective variable was coupled to its extended degree of freedom using a 10 kcal/mol/Å^2^ harmonic force constant; a 125 kcal/mol/Å^2^ force constant was used for Δ*RMSD*. Force constants were determined by trial-and-error as the lowest force constant sufficient to elicit the corresponding conformational change in short Steered Molecular Dynamics simulations. This ensures a coupling that is both strong enough to bias the dynamics, and soft enough to promote smooth exploration of the free energy surface [38]. Both extended degrees of freedom were thermostatted to 300 K with a Langevin thermostat with a 10 ps^−1^ friction constant. The *X*_*c*_ × Δ*RMSD* configurational space was discretized into a 0.75 Å×0.1 Å grid for the calculation. The *fullSamples* parameter was set to 2000 to limit non-equilibrium effects [63].

Similar to our previous work [26, 64], a two-step strategy was used to promote convergence of the eABF calculations. First, a 700 ns exploratory eABF run was performed starting from the equilibrated PR model, yielding near complete sampling of the configurational space. Second, the configurational space was divided into 12 non-overlapping windows separated by harmonic walls. Starting configurations for each window were extracted from the previous 700 ns run; each window was then simulated with eABF (starting with the gradient estimate obtained in the exploratory run) for 500 ns for a total of 6.7 μs of eABF simulation. This improves the sampling and refines the gradient estimate. Convergence was assessed as described in S1 Text, Section 1.

### String method in collective variables (CVSM) calculations

String method optimizations [31] with the “swarms-of-trajectories” variant [32] were used to compute MFEPs connecting the PR and PTS states. Strings were defined in either a 2-,12-, or 20-dimensional CV space designed to capture the essential features of the transition while remaining much simpler than full Cartesian space. The supporting CVs are listed in Table 1 along with their harmonic coupling force constant. The CVSM relaxes a discretized path in CV-space following the average drift computed from swarms of short MD simulations; up to unimportant dynamical effects, this average drift occurs along the direction of highest free energy gradient [32, 65, 66]. After each move, the string is reparametrized by enforcing equal spacing of the images, which projects out image displacement colinear to the string and ensures that string evolution takes place only along the orthogonal direction of the free energy gradient. Following standard practice, we reparametrize the string at each iteration by: 1) approximating the string by a piecewise linear interpolant; and 2) computing new image positions corresponding to equal spacing along the interpolant [31, 36]. Convergence is reached when orthogonal displacement is zero, that is, when the only image displacement is colinear to the string and thus, exactly cancelled out by reparametrization. The converged string then represents the MFEP between the end-states in the supporting CV-space [31]. Here, we assessed convergence by monitoring the RMSD in CV-space with respect to the initial string, according to current guidelines [67]. String calculations were performed using the Tcl scripting interface in NAMD 2.13 and the *colvars* module. Each CV was normalized between 0 and 1 by its total variation to ensure balanced reparametrization. Additional details on the string optimization procedure, parameters, and convergence analyses are reported in S1 Text, Section 2. Averaged, reparametrized strings over the last 50 iterations were considered for analysis. In total, our string calculations represent 2.3 μs of MD sampling.

### Umbrella sampling along the converged string

To gain insight into the free energy changes along the converged string A1, we performed Umbrella sampling simulations. First, we used linear interpolation to increase the resolution of the converged string from 32 to 128 equally-spaced images in normalized CV-space. Then, each image was simulated for 120 ns (total sampling 15.36 μs) under harmonic restraints applied to the same set of CVs and force constants as used for 12-dimensional string optimization, except that *χ*_11_ and *χ*_12_ were excluded from the set of supporting CVs. Umbrella sampling simulations were run with Gromacs 2021.5 patched with *colvars* [57]. For these calculations, the latest CHARMM36m force-field was used [68]. The comparison with results obtained with the CHARMM36 energy model is justified because differences between CHARMM36m and CHARMM36 mostly pertain to the description of intrinsically disordered proteins, which are unrelated to our study. The PMF along the string was computed using Umbrella Integration (UI) [39] with the chain rule as described in [64], see SI. 1- and 2-dimensional PMFs along arbitrary CVs were computed using the Multistate Bennett Acceptance Ratio (MBAR) method [40] with Gaussian kernel density estimation, as implemented in *pymbar* version 4.0 [41].

### Structural clustering of eABF simulations

To extract structural information about the metastable states sampled in eABF, we performed agglomerative hierarchical clustering with scikit-learn [69]. We used the RMSD over the CA atoms of the Relay and SH1 helices (after optimal translational and rotational fit) as distance metric. Numbers of clusters were chosen to ensure that at least 2 or 3 clusters with appreciable occupancy (> 10%) were obtained. This clustering approach was used to obtain representative frames for the metastables states sampled in the eABF simulation. For this purpose, we analyzed separately each metastable state as follows. First, we identified the stratified eABF frames belonging to the state, based on a simple geometrical criterion (S1 Text, Section 1). Then, we clustered these frames as described above. Finally, the representative structure was computed as the average structure of the most populated cluster. The representative structures of metastable states PTS1, I_*A*_, and I_*B*_ (shown on Fig 2B–2E) are available in Zenodo.

### Representative frames from umbrella sampling simulations

Representative frames used to detail the molecular mechanism of the PR → PTS transition were extracted from the US calculation along String A1 as follows. First, for each of the 128 images, the simulation frames were structurally aligned onto the CA atoms of the nearly-structurally-invariant N-ter subdomain (residues 50 to 170). Then, the average structure over all protein atoms was computed. The simulation frame closest to the average (in least-RMSD sense, considering the CA atoms of Switch II, Relay helix, Relay loop, SH2 helix, SH1 helix, and converter, *i.e.,* residues 457 to 512 and 683 to 789, for RMSD calculation) was used as representative (or “typical”) frame for the corresponding image. The 128 typical frames along the full path are available in Zenodo, and were used for the preparation of S6a Fig. We retained the typical frames of images 13 (PR), 30 (I_*A*_), 74 (I_*A*_2) and 111 (PTS1) to prepare Fig 3C–3J. The typical frame of image 116 (also belonging to PTS1) was used to prepare Fig 4.

## Supporting information

S1 Text Supporting analyses. Convergence, error analysis and metastable state analysis for eABF calculations; String method calculations; Free energy profiles along the string from umbrella sampling; Mechanism along Path B.
(PDF)

S1 Table Component-wise and total converter swing in the myosin VI recovery stroke. (PDF)

S2 Table Elliptical regions defining metastable states, used in the analysis of eABF simulations. (PDF)

S3 Table CVSM simulations in 2D CV space. (PDF)

S4 Table CVSM simulations in 12D CV space. (PDF)

S5 Table CVSM simulation in 20D CV space. (PDF)

S1 Fig Sampling of the configurational space in eABF simulations. The number of counts, *i.e.*, the number of times a given grid point has been visited, is shown in logarithmic scale. Black dotted lines indicate the window boundaries in stratified simulation. Window labels are shown.
(TIF)

S2 Fig Convergence of generalized force estimate in eABF simulations. (TIF)

S3 Fig Statistical error estimate for the free energy landscape from bootstrap-like analysis (in kcal mol^−1^). (TIF)

S4 Fig eABF calculations sample open Switch II conformers consistent with independent PTS simulations. (a) Distances *d*_1_ and *d*_*γ*_ never sample the closed Switch II region in stratified eABF simulations. Shown are *d*_1_, *d*_*γ*_ scatter plots colored per window. (b) The PTS basin identified in eABF calculation is consistent with independent, unbiased MD simulations of Myo6. Shown are density lines for *X*_*c*_ and Δ*RMSD* from the Myo6 simulations we reported in [26]. Of note are: the remarkable agreement of the PTS unbiased simulations with the free energy landscape; the sampling of a converter movement in simulation PR+ATP (3) which is roughly consistent with metastable state I_*A*_; and the clear separation of PTS from PPS.
(TIF)

S5 Fig Statistical distribution of selected observables in substates identified in eABF calculations. (a) Distribution of converter coordinates *X*_*c*_ and *Y*_*c*_. (b) Distribution of Relay helix backbone hydrogen bonds *k*_3_ and *k*_5_. The difference in converter position between I_*A*_ and I_*B*_, along with the atypical hydrogen bonding pattern of the Relay helix in I_*B*_, are consistent with I_*A*_ and I_*B*_ not belonging to the same transition pathway.
(TIF)

S6 Fig The same I_*A*_ intermediate is captured in eABF and string/US calculations. (a) RMSD of structures sampled along String A1 with respect to the representative structure of I_*A*_ sampled in eABF, computed after optimal fit on the CA atoms of the Relay and SH1 helices. The RMSD clearly is minimum and below 1 Å when the progress along the path is roughly between 0.24 and 0.35, which corresponds to the I_*A*_ basin as identified in string/US calculations. The red dot marks the minimal RMSD value, achieved at image 36 along the string. (b) Structural comparison of the Relay helix, SH1 helix and converter between the representative structure of state I_*A*_ sampled in eABF (cyan) and in string/US (image 36, red). The conformation and orientation of the structural elements are extremely close. These results demonstrate that the same metastable basin is sampled in both independent calculations, which justifies referring to them as I_*A*_ regardless of their provenance. The representative frame of I_*A*_ from eABF is the same as shown on Fig 2c. The typical umbrella sampling frames along string A1 (see Methods) were used to compute the RMSD profile in panel a; among them, frame 36 was used to produce panel b.
(TIF)

S7 Fig CVSM calculations in 2D CV-space are consistent with the eABF free energy landscape. (TIF)

S8 Fig Convergence of CVSM calculations in 2D CV-space. Shown is the RMSD in normalized CVs from the initial path. The greyed area shows the last 50 iterations, over which the average strings pictured in S7 Fig are computed.
(TIF)

S9 Fig Convergence of CVSM calculations in 12D CV-space. Shown is the RMSD in normalized CVs from the initial path. The greyed area shows the last 50 iterations, over which the average strings are computed.
(TIF)

S10 Fig Projections of the 12D converged strings onto 2D space reveal two alternate pathways lying within the transition tube predicted by eABF. (TIF)

S11 Fig Evolution of biased structural observables along converged, averaged Strings A1 and A2. (TIF)

S12 Fig Contacts between the N-terminal subdomain and the converter during the PR→ PTS transition. (a-d) Close-up on the converter/N-ter interface in representative frames of the metastable states sampled in umbrella sampling along String A1. Residues involved in contacts are shown as sticks. For clarity, non-polar hydrogens are not shown. (e) PMF along *X*_*c*_ and the number of contacts estimated with MBAR from umbrella sampling along string A1. A contact is defined as two heavy atoms being closer than 4.5 Å. Frames are the same as the ones from Fig 3.
(TIF)

S13 Fig Evolution of biased structural observables along converged, averaged Strings B1 and B2. (TIF)

S14 Fig A secondary kink in the Relay helix forms in Path B. (a) Initial state (PR-like): the Relay helix is straight and intact, the converter interacts with the N-terminal subdomain. (b) *α* = 0.25: a secondary kink in the Relay helix (red arrow) accommodates converter movement. The converter is still in contact with the N-terminal subdomain. (c) Final state (PTS-like): the converter has moved and broken the contacts, and the canonical kink (red arrow) in the Relay helix has formed. Shown are the last simulation frames of the last iteration of string B1 optimization, for images indicated at the bottom of each panel. The complete sequence of structures along string B1 is available in Zenodo.
(TIF)

S15 Fig Re-optimization of String B1 in 20-dimensional space. (a) Convergence of the CVSM calculation in 20D CV-space. (b-f) Evolution of converter/N-terminal distances along the converged, averaged 20D string. (g-i) Evolution of the RH backbone distances describing the secondary kink along the converged, averaged 20D string. Panels b and c show that contacts between the converter and the N-terminal subdomain are preserved until about halfway along the transition, while panels g-i show that a secondary kink forms in the RH from about 0.25 to 0.50 progress along the string.
(TIF)
