# Supplementary material for: A weak coupling mechanism for the early steps of the recovery stroke of myosin VI: A free energy simulation and string method analysis
Source: PLoS Comput Biol. 2024 Apr 25;20(4):e1012005. doi: 10.1371/journal.pcbi.1012005 (PMC11086841; doi:10.1371/journal.pcbi.1012005)
Supplement: S1 Text — Convergence, error analysis and metastable state analysis for eABF calculations; String method calculations; Free energy profiles along the string from umbrella sampling; Mechanism along Path B. (PDF) [file pcbi.1012005.s001.pdf]

# Supporting Information - A weak coupling mechanism for the early steps of the recovery stroke of myosin VI: a free energy simulation and string method analysis

Florian E.C. Blanc [1,2,#,*], Anne Houdusse [2], Marco Cecchini [1,*]

**1** Molecular Function Engineering, Institut de Chimie de Strasbourg, UMR 7177 CNRS, Université de Strasbourg, Strasbourg, France
**2** Structural Motility, Institut Curie, CNRS, UMR 144, PSL Research University, Paris, France

# Current Address: Biophysics of Complex Systems, Institut des Sciences Analytiques, UMR 5280, Université Claude Bernard Lyon 1, CNRS, Villeurbanne, France
* florian.blanc@normalesup.org * mcecchini@unistra.fr

# S1 Text: Supporting analyses

## 1 Convergence, error analysis and metastable state analysis for eABF calculations

As seen on S1 Fig, all relevant regions of configurational space are visited multiple times by the eABF simulations. Convergence of eABF calculations was assessed by monitoring the per-window RMSD of the free energy gradient estimate (S2 Fig). Most windows representative of the transition tube ($W_{11}, W_{21}, W_{22}, W_{32}, W_{33}, W_{43}$) show a plateau close to $0.5\,\mathrm{kcal\,mol^{-1}\,\AA^{-1}}$ RMSD over the last few tens of ns, suggesting that the generalized force estimate has converged. Bootstrap-like statistical error analysis was performed as in our previous work [1, 2] and shows that statistical error on the free energy values is small ($< 1.0\,\mathrm{kcal\,mol^{-1}}$) everywhere in the relevant regions of 2D free energy landscape, and does not go above $2.0\,\mathrm{kcal\,mol^{-1}}$ even at the irrelevant margins (S3 Fig). The statistical error at the first free energy barrier is of order $1.0\,\mathrm{kcal\,mol^{-1}}$.

From the stratified eABF simulation, ensembles of frames belonging to identified metastable states were determined as follows. First, each state was defined geometrically in $(X_c, \Delta RMSD)$ as an elliptical region centered on the position of the local free energy minimum, and whose axes were chosen to obtain reasonable coverage of the free energy basin. The center and axis lengths of the ellipses defining states $\mathrm{I}_A$, $\mathrm{I}_B$ and PTS1 are reported in S2 Table. The ensembles of structures describing each state were used to compute the state-specific statistical distributions of observables reported on S5 Fig, and were analyzed by clustering to obtain representative structures shown on Figs 2 and S6.

## 2 String method calculations

### Procedure

An iteration of the string method with swarms of trajectories consists of the following operations, performed for each image:

1. Equilibration of length $t_{eq}$ of the system around its image along the string in CV-space using harmonic restraining potentials.
2. Parallel, unrestrained simulation of $n_{swarm}$ replicas for $t_{free}$ initiated from the equilibrated system

For each image, the average drift in CV-space measured over the swarm of unrestrained trajectories is used to update the string. Then, the string is smoothed by local averaging, and reparametrized [3, 4]. This process is iterated until convergence is reached. Convergence of string optimizations is assessed by monitoring the Root Mean Square Deviation (RMSD) of the string in normalized CV-space with respect to the initial string. The string is considered converged when this value stabilizes. String optimization is akin to a gradient-based high-dimensional optimization, for which a well-known issue is the existence of multiple locally optimal solutions. A careful choice of the initial "guess" path from which string iterations are initialized mitigates this issue by making it more likely to converge to a biologically meaningful solution. Comparing converged strings from different guess path initializations also allows one to assess the robustness of the findings. For these reasons, we consider several different guess paths in our string calculations.

## String optimizations in $(X_c,\ \Delta RMSD)$ space are consistent with eABF results

To validate the transition tube predicted by eABF, we first performed string optimizations in $(X_c,\ \Delta RMSD)$-space.

### Stability of the eABF-predicted MFEP under explicit CVSM dynamics

First, we sought to assess whether the MFEP computed analytically over the eABF free energy landscape were stable under explicit (*i.e.*, atomistic) CVSM dynamics. We constructed this path (hereafter eABF-MFEP) by 1) evaluating analytical derivatives of the eABF free energy landscape and 2) relaxing a 2D string on this free energy landscape using the zero-temperature string method [5] in normalized coordinates. These operations were implemented in a Python/Scipy script [6, 7]. Then, we prepared equilibrated Myo6 conformers along this path in $(X_c,\ \Delta RMSD)$-space as follows. We "pulled" Myo6 (from the equilibrated PR model) along the eABF-predicted MFEP using a 120 ns-long Steered MD simulation on $X_c$ and $\Delta RMSD$ with the harmonic force constants reported in Table 1. 32 conformers equally-spaced in normalized CV-space were extracted from the Steered MD trajectory and further equilibrated by 20 ns of umbrella sampling with the same force constants. The resulting conformers were then used to run 2 separate CVSM calculations in 2D space (S3 Table). In both cases, the path obtained by averaging the strings over the last 50 iterations is remarkably close to the eABF-predicted MFEP. Therefore, up to equilibrium fluctuations, the 2D transition path predicted by eABF calculations is essentially invariant under the string method dynamics, independently confirming that it lies close to the MFEP (S7 and S8 Figs).

### Relaxation of the straight guess path towards the eABF-predicted MFEP under explicit CVSM dynamics

Using a similar protocol, we prepared equilibrated Myo6 conformers equally-spaced along the straight path joining PR and PTS in 2D CV-space. Relaxation of this straight guess path by two independent CVSM runs shows that it consistently evolves towards the eABF-predicted transition tube (S7 Fig and S3 Table). We conclude that

independent string optimizations in 2D space confirm the important features of the transition mechanism predicted by eABF.

## String method calculations in 12D normalized CV-space

### Generation of a 12-dimensional guess path from the 2D eABF-MFEP by uplifting

The eABF-MFEP is representative of the general features of the PR → PTS transition, but the 2D CV-space is too coarse-grained to capture the finer details of the mechanism. The 2D MFEP and surrounding transition tube correspond to projections of a set of transition pathways lying in a space of higher dimensionality. Refined approximations of the true MFEP could be obtained by backmapping, or ”uplifting”, the 2D MFEP into a higher-dimensional space provided that the new coordinates offer a better descriptive power of the transition mechanism, that is, if they capture relevant aspects of the transitions overlooked by the coarser description. We developed a method to uplift the 2D eABF-MFEP by embedding it into a 12D CV-space designed to provide a higher-resolution representation of the recovery stroke by 1) including additional degrees of freedom and 2) replacing the coarse-grained $\Delta RMSD$ Relay helix kink descriptor by 4 separate distance CVs representing local backbone rearrangements upon the formation of the kink. This uplifted string provides the starting point for a new series of CVSM calculations, this time in 12D space.

Such an uplifting requires accounting for the missing information of the additional degrees of freedom. Here, we used the average values of the additional CVs measured from the pre-equilibration Umbrella Sampling simulations along the 2D eABF-MFEP reported above. These provide reasonable initial estimates of the most likely value taken by the additional CVs when the system is restrained in the vicinity of the MFEP. However, the resulting path in 12D CV-space was found to be very rugged, which we reasoned would impede convergence of the string calculations. To alleviate this issue, we regularized the uplifted path by averaging it with a straight path connecting PR to PTS in normalized 12-dimensional CV space. The resulting uplifted regularized path retains the global shape of the uplifted path before regularization, but is much smoother.

### CVSM calculations from the regularized uplifted string

The 32 Myo6 conformers previously pre-equilibrated in 2D along the eABF-MFEP were further relaxed along the regularized uplifted string in 12D by 1 ns of US. Then, they were used to perform two independent CVSM calculations to reveal the MFEP in 12D CV-space, yielding Strings A1 and A2.

### 12D CVSM calculations from the straight guess path

To assess the robustness of the string calculations, we also ran independent string calculations initiated from the linear guess path connecting PR to PTS in normalized 12-dimensional CV-space. 32 equally-spaced conformers along this path were prepared by relaxing the conformers previously pre-equilibrated along the 2D linear path to their corresponding images in the 12D linear string using 1 ns of US. Then, they were used to perform two separate CVSM calculations, yielding Strings B1 and B2. The corresponding transition path, or Path B, is described in S1 Text, Section 4.

### String method calculations in 20D normalized CV-space

The analysis of the transition pathway emerging from the 12D string optimizations initialized with the straight guess path (*i.e.*, Strings B1 and B2) revealed 8 additional potentially relevant degrees of freedom, see S1 Text, Section 4. To better reflect their involvement in the mechanism, we uplifted and regularized the converged String B1 to the 20-dimensional CV-space using the procedure described above. The 20D regularized/uplifted string was then relaxed to convergence by 39 iterations of CVSM.

### Projection of the high-dimensional strings onto 2D $(X_c,\ \Delta RMSD)$-space

We projected the high-dimensional (12 or 20 dimensions) strings onto 2D $(X_c,\ \Delta RMSD)$-space to compare them with the eABF results. Since the $\Delta RMSD$ CV is not a component of the high-dimensional strings, we evaluated its value as the per-image average over the last 50 string iterations. Since $\Delta RMSD$ is not explicitly biased nor involved in string reparametrization, the projected strings have a rugged aspect.

## 3 Free energy profiles along the string from umbrella sampling

### Umbrella sampling

An "on-the-path" Umbrella Sampling calculation was performed along the averaged string from the first string optimization initiated from the eABF-regularized guess, or String A1. First, $\chi_{11}$ and $\chi_{12}$ were excluded from the set of biased CVs because we reasoned that the sharp nature of rotameric transitions could hinder sampling around the transition state regions. Then, we linearly interpolated each CV along the string using Scipy. Finally, 128 images equally-spaced along the string in normalized CV space were extracted and used as restraint centers for umbrella sampling. Umbrella sampling simulations were run with GROMACS 2021.5 patched with *colvars*, using the same force constants as for string optimizations, and were initiated from structures sampled in the last iteration of the string optimization. To equilibrate the images with the restraining potentials, the first 1 ns were discarded for each window. Each window was then simulated for 120 ns, resulting in a total of 15.36 µs of umbrella sampling simulation. For analysis, CV data were saved every 10 ps. Free energies along the string were then computed using two different approaches.

### Free energy profile along the string by umbrella integration

To obtain a PMF along the string, we used the procedure described in [8]. Introducing $\alpha$ as a non-dimensional progress variable along the path, such that $\alpha = 0$ at the beginning of the path and $\alpha = 1$ at the end, one can write for the free energy $F(\alpha)$ along the path:

$$\frac{\mathrm{d}F(\alpha)}{\mathrm{d}\alpha} = \sum_i \frac{\partial F}{\partial \xi_i} \cdot \frac{\mathrm{d}\xi_i}{\mathrm{d}\alpha} \tag{S1}$$

where $\xi_i$ refers to CV $i$ along the string, and the sum runs over all supporting CVs. The $\frac{\mathrm{d}\xi_i}{\mathrm{d}\alpha}$ are evaluated by analytical differentiation of fitted B-splines. To estimate the free energy gradients with respect to CV components, we used the Umbrella

Integration estimator [9] with a number of bins equal to the number of windows, *i.e.* 128.

### MBAR reweighting

Separately from the UI calculations, two-dimensional free energy profiles along arbitrary CVs (possibly including unrestrained ones) were computed using the Multistate Bennett Acceptance Ratio (MBAR) method [10]. These PMFs represent the effective free energy landscapes when the progress coordinate along the PR → PTS transition is averaged out, and provide insight into the thermodynamic coupling between elementary rearrangements. MBAR calculations were performed using *pymbar* 4.0.1. We used the recently published Kernel Density Estimation (KDE)-based procedure to obtain smooth free energy estimates [11]. In so doing, we found that the choice of the KDE bandwidth parameter could strongly affect the value of the free energy barriers, but only marginally affected the position and relative stability of free energy basins. Therefore, the MBAR-computed free energy maps should be considered semi-quantitative.

## 4 Mechanism along Path B

From the straight guess path, two independent string optimizations were performed until they converged towards a common sequence of events, which we name Path B (S9 Fig). Similar to the situation for Path A, Path B from string calculations is virtually identical to the Path $B_{ABF}$, as we justify below. Projection of the 2 Path B 12D-strings onto the ($X_c$, $\Delta RMSD$) plane shows that they are consistent with the shape and location of the transition tube (S10 Fig). Interestingly, Paths A and B do not overlap, and Path B visits $I_B$. Thus, even if they share the same gross features, the structural mechanism predicted from Path B is a bit different from that of Path A (S13 Fig). In Path B, the kink in the RH forms with a different timing and mechanism. There, the initial movement of the converter is not accommodated by the disruption of its interactions with the N-terminal subdomain, but by the transient formation of a secondary kink in the RH at the level of residues 490 to 496 (S14 Fig). This is similar to Path $B_{ABF}$ from eABF calculations (see notably Fig 2E) and supports the idea that Paths $B_{ABF}$ and $B$ are in fact representative of the same mechanism. As the hydrogen-bonding distances describing the formation of the secondary kink are not explicitly biased in the 12D string optimizations, it is difficult to know whether the mechanism observed in Path B does correspond to an MFEP also along these degrees of freedom. To clarify this matter, we extended the CV-space to 20 dimensions by adding 3 distances describing the secondary kink and 5 distances describing converter/N-ter interactions, uplifted the first converged Path B string to this space, and relaxed it with the string method (S1 Text, Section 2, and S15A Fig). The resulting pathway still exhibits the same sequence of events, supporting its relevance as a locally optimal MFEP in the PR → PTS transition.

The extended, highly bent configuration of the Relay helix enables the rotation of the converter while some of its contacts with the N-terminal subdomain are preserved (S15B-I and S14 Figs). This configuration corresponds to the $I_B$ basin identified in eABF, which may represent a metastable intermediate state along this path. Next, upon going from $I_B$ to PTS1, this secondary kink disappears and the canonical kink in the RH forms concurrently with continued movement of the converter in a fashion similar to Path A.

The sensitivity to the initial guess of the converged path is not overly surprising, as multiple pathways are expected for a system the size of myosin's even after reducing

its dimensionality to 12 (or 20). It is plausible that both families of pathways may actually be explored in the recovery stroke of Myo6. Thus, the Relay helix exhibits significant plasticity throughout its length, which may allow it to bend significantly and even develop transient kinks to accomodate the extensive movement of the converter through a diversity of mechanisms. Nevertheless, Path A is likely to be more representative of the dominant mechanism because it entails less structural perturbation (*i.e.,* only one kink forms), which arguably is a desirable property for an MFEP.

## References


1. Blanc F, Isabet T, Benisty H, Sweeney HL, Cecchini M, Houdusse A. An Intermediate Along the Recovery Stroke of Myosin VI Revealed by X-ray Crystallography and Molecular Dynamics. Proceedings of the National Academy of Sciences. 2018; p. 201711512. doi:10.1073/pnas.1711512115.

2. Wereszczynski J, McCammon JA. Nucleotide-Dependent Mechanism of Get3 as Elucidated from Free Energy Calculations. Proceedings of the National Academy of Sciences. 2012;109(20):7759–7764.

3. Maragliano L, Fischer A, Vanden-Eijnden E, Ciccotti G. String Method in Collective Variables: Minimum Free Energy Paths and Isocommittor Surfaces. The Journal of Chemical Physics. 2006;125(2):024106. doi:10.1063/1.2212942.

4. Pan AC, Sezer D, Roux B. Finding Transition Pathways Using the String Method with Swarms of Trajectories. The Journal of Physical Chemistry B. 2008;112(11):3432–3440. doi:10.1021/jp0777059.

5. E W, Ren W, Vanden-Eijnden E. String Method for the Study of Rare Events. Physical Review B. 2002;66(5). doi:10.1103/PhysRevB.66.052301.

6. Jones E, Oliphant T, Peterson P. {SciPy}: Open Source Scientific Tools for {Python}. 2001–///;.

7. Virtanen P, Gommers R, Oliphant TE, Haberland M, Reddy T, Cournapeau D, et al. SciPy 1.0: Fundamental Algorithms for Scientific Computing in Python. Nature Methods. 2020;17(3):261–272. doi:10.1038/s41592-019-0686-2.

8. Blanc FE, Cecchini M. An Asymmetric Mechanism in a Symmetric Molecular Machine. The Journal of Physical Chemistry Letters. 2021;12(13):3260–3265. doi:10.1021/acs.jpclett.1c00404.

9. Kästner J, Thiel W. Bridging the Gap Between Thermodynamic Integration and Umbrella Sampling Provides a Novel Analysis Method: “Umbrella Integration”. The Journal of Chemical Physics. 2005;123(14):144104. doi:10.1063/1.2052648.

10. Shirts MR, Chodera JD. Statistically Optimal Analysis of Samples from Multiple Equilibrium States. The Journal of Chemical Physics. 2008;129(12):124105. doi:10.1063/1.2978177.

11. Shirts MR, Ferguson AL. Statistically Optimal Continuous Free Energy Surfaces from Biased Simulations and Multistate Reweighting. Journal of Chemical Theory and Computation. 2020;16(7):4107–4125. doi:10.1021/acs.jctc.0c00077.
