# Supplementary material for: A weak coupling mechanism for the early steps of the recovery stroke of myosin VI: A free energy simulation and string method analysis
Source: PLoS Comput Biol. 2024 Apr 25;20(4):e1012005. doi: 10.1371/journal.pcbi.1012005 (PMC11086841; doi:10.1371/journal.pcbi.1012005)
Supplement: S1 Table — (PDF) [file pcbi.1012005.s002.pdf]

Structure	X_c	Y_c	Z_c	ΔX_c	ΔY_c	ΔZ_c	ΔR_c	%total
PR (x-ray)	4.66	-7.12	53.8	0	0	0	0	0
PPS (x-ray)	-17.1	-0.474	46.5	-21.8	6.6	-7.3	23.9	100
PTS (x-ray)	-10.5	-4.86	53.2	-15.2	2.3	-0.6	15.3	64.2
A1 image 31	-9.78	-4.4	52.9	-14.4	2.7	-0.9	14.7	61.6

S1 Table: Component-wise and total converter swing in the myosin VI recovery stroke. All swing values are in Å. The component-wise swing along X_c from PR to a given structure is defined as $X_c(\text{structure}) - X_c(PR)$. Other components are defined similarly. The total swing is defined as $\Delta R_c = \sqrt{\Delta X_c^2 + \Delta Y_c^2 + \Delta Z_c^2}$. The percentage of total swing is equal to $100 \times \Delta R_c(\text{structure})/\Delta R_c(PPS)$.
