# Supplementary material for: A weak coupling mechanism for the early steps of the recovery stroke of myosin VI: A free energy simulation and string method analysis
Source: PLoS Comput Biol. 2024 Apr 25;20(4):e1012005. doi: 10.1371/journal.pcbi.1012005 (PMC11086841; doi:10.1371/journal.pcbi.1012005)
Supplement: S2 Table — (PDF) [file pcbi.1012005.s003.pdf]

State	$(X_c, \Delta RMSD)$ center	$(X_c, \Delta RMSD)$ diameters
I_A	-3.8 Å, 1.025 Å	2 Å, 0.25 Å
I_B	-2.5 Å, 0.38 Å	3 Å, 0.3 Å
PTS1	-6.2 Å, -0.6 Å	3.2 Å, 0.25 Å

S2 Table: Elliptical regions defining metastable states, used in the analysis of eABF simulations.
