# Supplementary material for: A weak coupling mechanism for the early steps of the recovery stroke of myosin VI: A free energy simulation and string method analysis
Source: PLoS Comput Biol. 2024 Apr 25;20(4):e1012005. doi: 10.1371/journal.pcbi.1012005 (PMC11086841; doi:10.1371/journal.pcbi.1012005)
Supplement: S3 Table — (PDF) [file pcbi.1012005.s004.pdf]

Calculation	Guess path	Ends	t_{eq} (ps)	n_{swarm}	t_{free} (ps)	n_{iter}	Total (ns)
2D String (1)	eABF-MFEP	Fixed	1	10	0.5	201	38.6 ns
2D String (2)	eABF-MFEP	Fixed	1	10	0.5	116	22.3 ns
2D String (3)	Straight	Free	1	10	0.5	127	24.4 ns
2D String (4)	Straight	Fixed	1	10	1	119	41.9 ns

S3 Table: CVSM simulations in 2D CV space.
