# Supplementary material for: A weak coupling mechanism for the early steps of the recovery stroke of myosin VI: A free energy simulation and string method analysis
Source: PLoS Comput Biol. 2024 Apr 25;20(4):e1012005. doi: 10.1371/journal.pcbi.1012005 (PMC11086841; doi:10.1371/journal.pcbi.1012005)
Supplement: S4 Table — (PDF) [file pcbi.1012005.s005.pdf]

Calculation	Guess path	Ends	t_{eq} (ps)	n_{swarm}	t_{free} (ps)	n_{iter}	Total (ns)
12D String A1	uplifted/regularized eABF-MFEP	Fixed	100	20	0.5	179	630 ns
12D String A2	uplifted/regularized eABF-MFEP	Fixed	100	20	0.5	119	418.9 ns
12D String B1	Straight (12D)	Fixed	100	20	0.5	199	700.5 ns
12D String B2	Straight (12D)	Fixed	100	20	0.5	119	418.9 ns

S4 Table: CVSM simulations in 12D CV space
