# Supplementary material for: A weak coupling mechanism for the early steps of the recovery stroke of myosin VI: A free energy simulation and string method analysis
Source: PLoS Comput Biol. 2024 Apr 25;20(4):e1012005. doi: 10.1371/journal.pcbi.1012005 (PMC11086841; doi:10.1371/journal.pcbi.1012005)
Supplement: S5 Table — (PDF) [file pcbi.1012005.s006.pdf]

Calculation	Guess path	Ends	t_{eq} (ps)	n_{swarm}	t_{free} (ps)	n_{iter}	Total (ns)
20D String (1)	Averaged converged string from 12D String B1 uplifted/regularized to 20D space	Fixed	1	10	0.5	39	7.5 ns

S5 Table: CVSM simulation in 20D CV space.
