# Supplementary figures and images for: A weak coupling mechanism for the early steps of the recovery stroke of myosin VI: A free energy simulation and string method analysis
Source: PLoS Comput Biol. 2024 Apr 25;20(4):e1012005. doi: 10.1371/journal.pcbi.1012005 (PMC11086841; doi:10.1371/journal.pcbi.1012005)

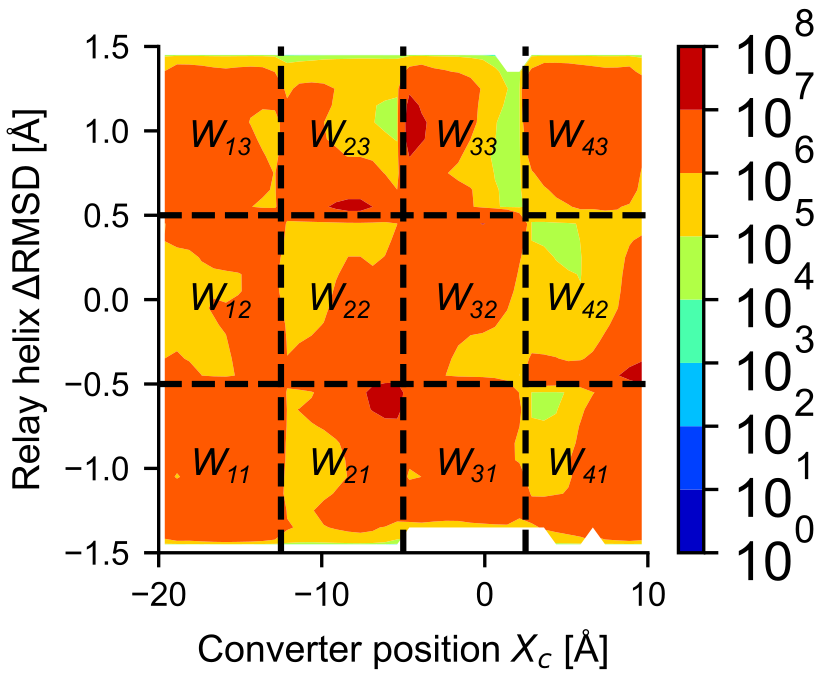

Supplement: S1 Fig — The number of counts, i.e., the number of times a given grid point has been visited, is shown in logarithmic scale. Black dotted lines indicate the window boundaries in stratified simulation. Window labels are shown. (TIF) [file pcbi.1012005.s007.tif]

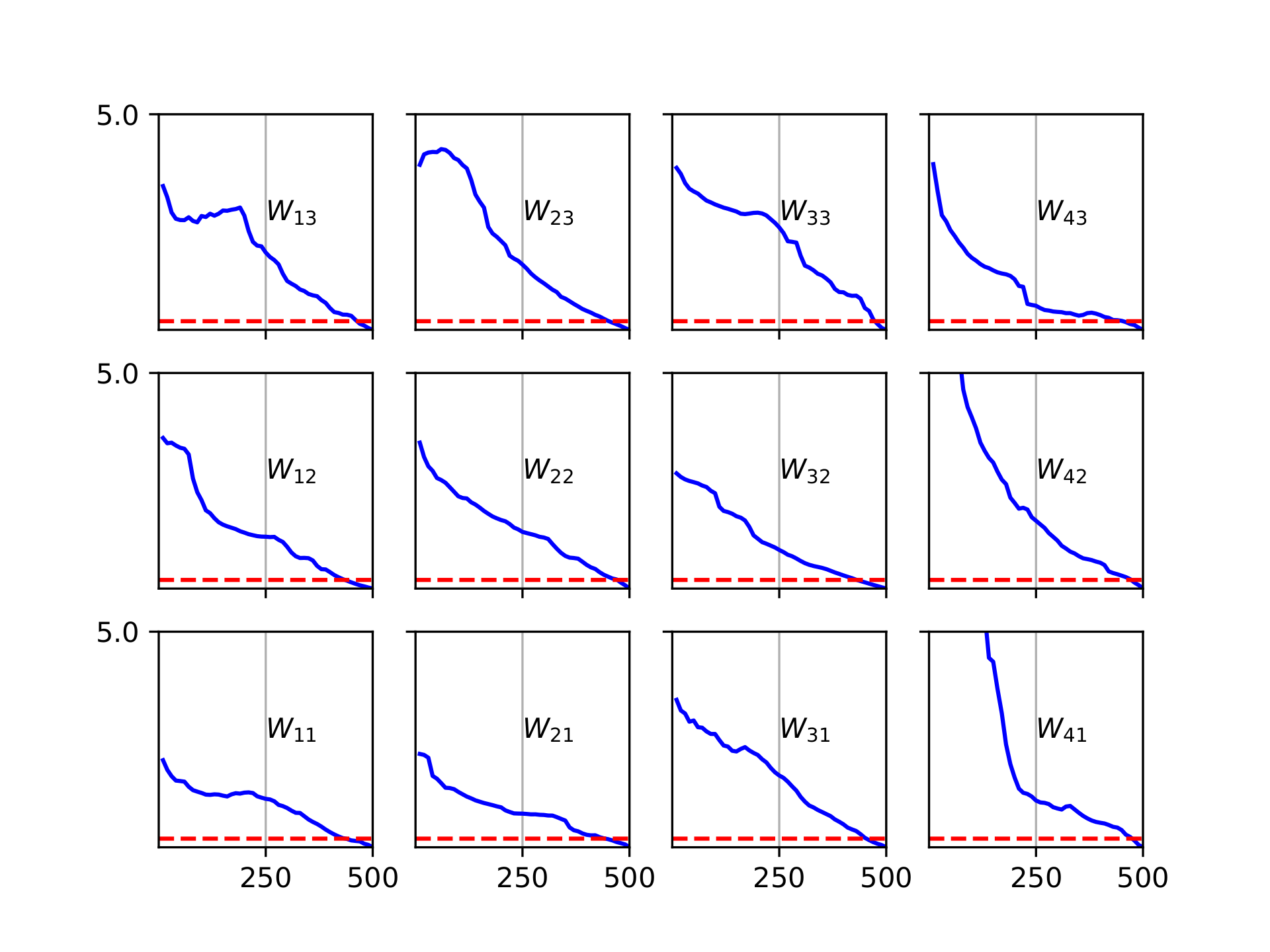

Supplement: S2 Fig — (TIF) [file pcbi.1012005.s008.tif]

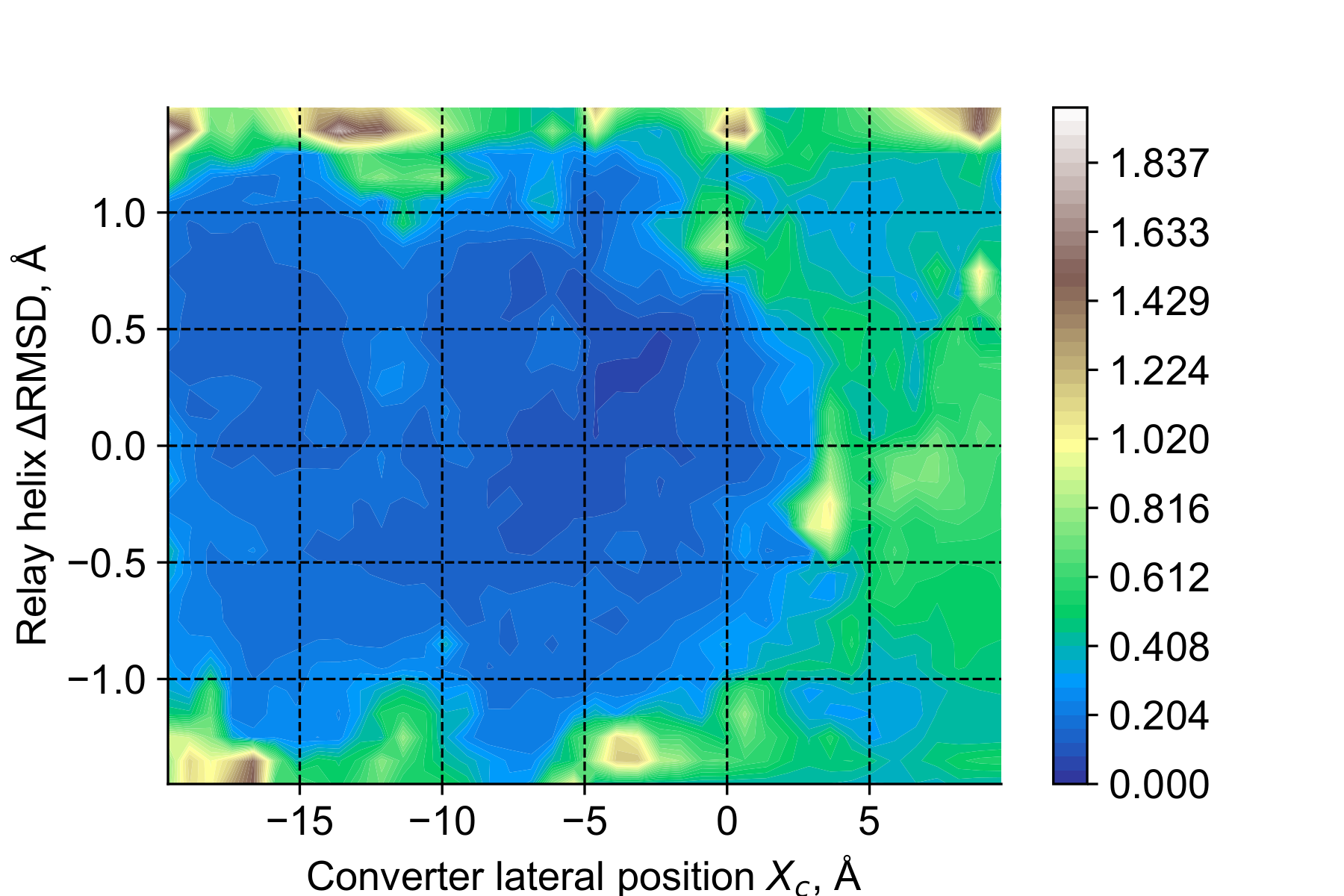

Supplement: S3 Fig — (TIF) [file pcbi.1012005.s009.tif]

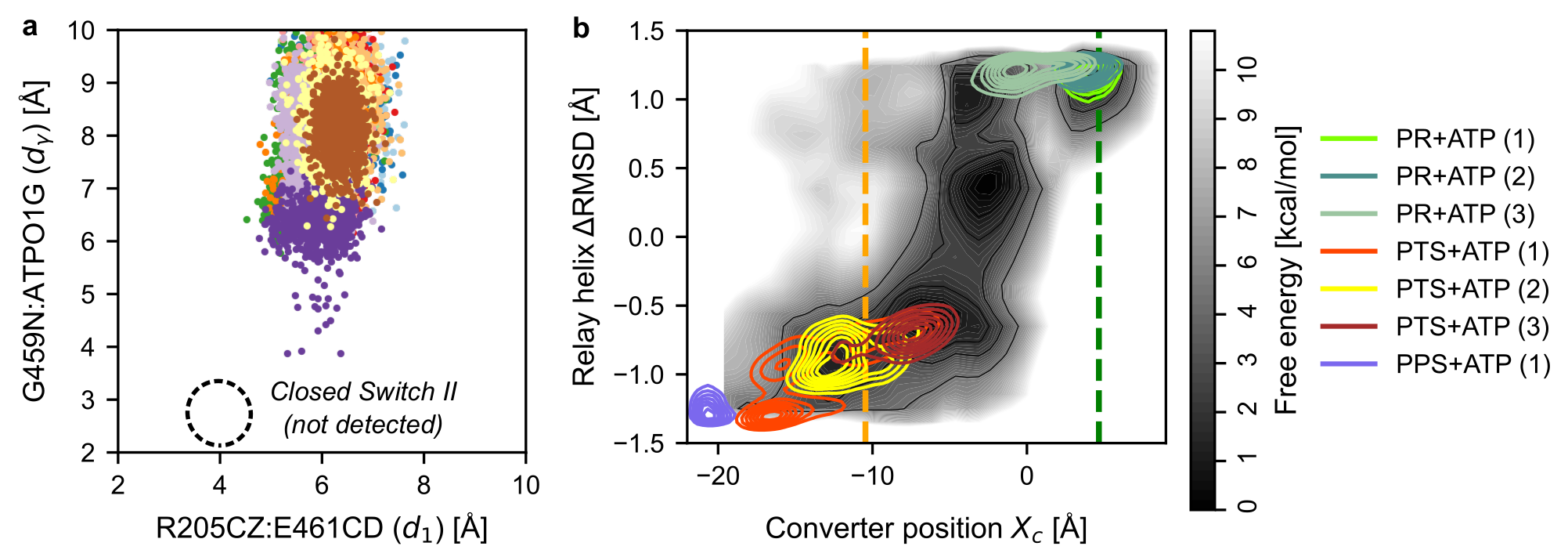

Supplement: S4 Fig — (a) Distances d1 and dγ never sample the closed Switch II region in stratified eABF simulations. Shown are d1, dγ scatter plots colored per window. (b) The PTS basin identified in eABF calculation is consistent with independent, unbiased MD simulations of Myo6. Shown are density lines for Xc and ΔRMSD from the Myo6 simulations we reported in [26]. Of note are: the remarkable agreement of the PTS unbiased simulations with the free energy landscape; the sampling of a converter movement in simulation PR+ATP (3) which is roughly consistent with metastable state IA; and the clear separation of PTS from PPS. (TIF) [file pcbi.1012005.s010.tif]

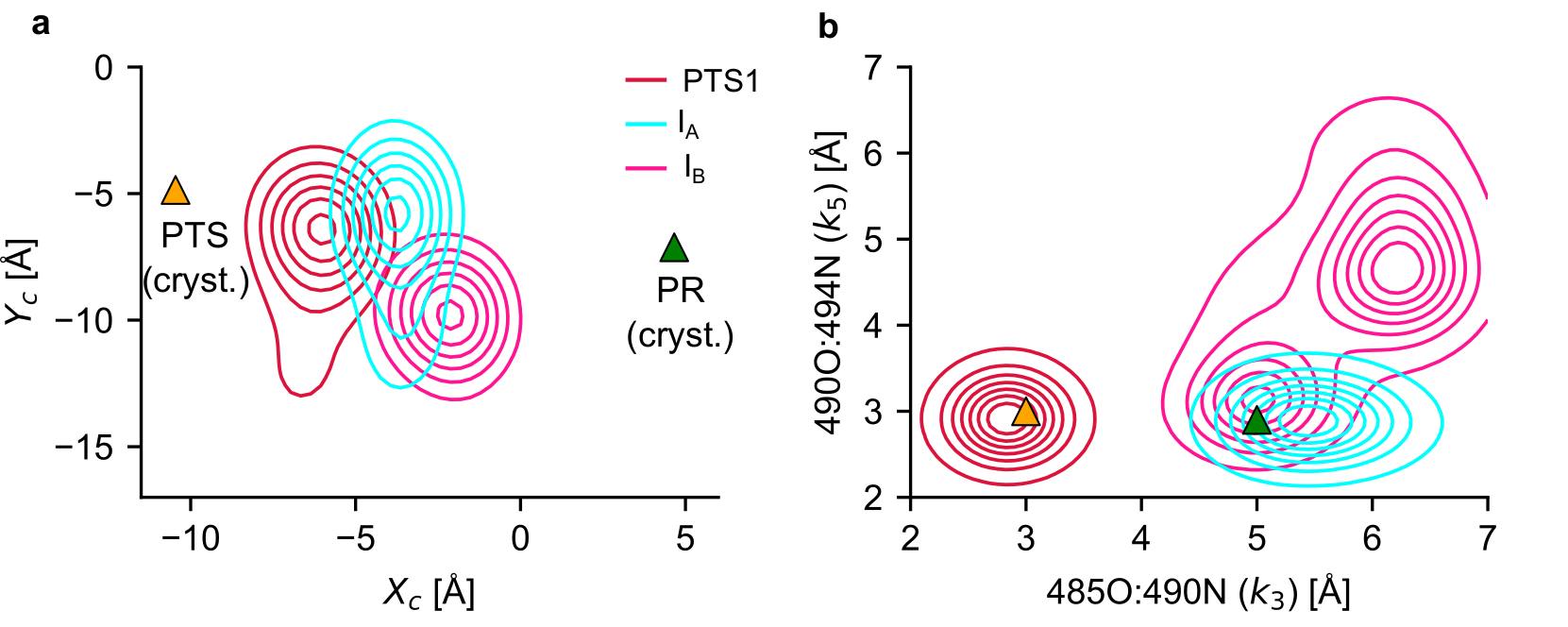

Supplement: S5 Fig — (a) Distribution of converter coordinates Xc and Yc. (b) Distribution of Relay helix backbone hydrogen bonds k3 and k5. The difference in converter position between IA and IB, along with the atypical hydrogen bonding pattern of the Relay helix in IB, are consistent with IA and IB not belonging to the same transition pathway. (TIF) [file pcbi.1012005.s011.tif]

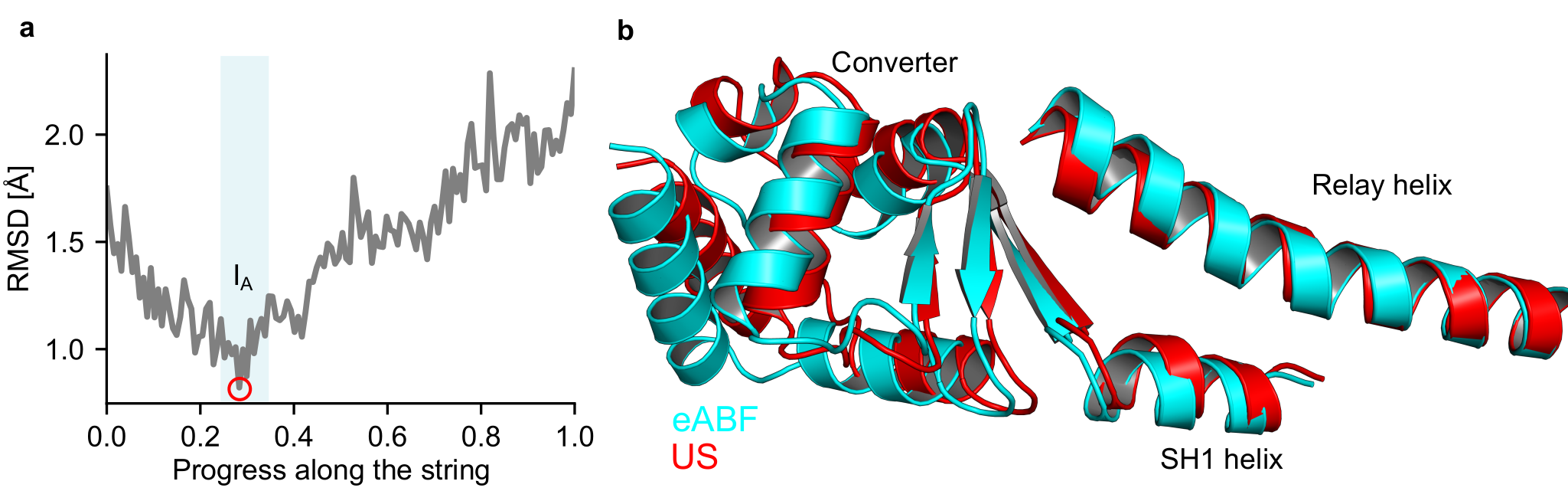

Supplement: S6 Fig — (a) RMSD of structures sampled along String A1 with respect to the representative structure of IA sampled in eABF, computed after optimal fit on the CA atoms of the Relay and SH1 helices. The RMSD clearly is minimum and below 1 Å when the progress along the path is roughly between 0.24 and 0.35, which corresponds to the IA basin as identified in string/US calculations. The red dot marks the minimal RMSD value, achieved at image 36 along the string. (b) Structural comparison of the Relay helix, SH1 helix and converter between the representative structure of state IA sampled in eABF (cyan) and in string/US (image 36, red). The conformation and orientation of the structural elements are extremely close. These results demonstrate that the same metastable basin is sampled in both independent calculations, which justifies referring to them as IA regardless of their provenance. The representative frame of IA from eABF is the same as shown on Fig 2c. The typical umbrella sampling frames along string A1 (see Methods) were used to compute the RMSD profile in panel a; among them, frame 36 was used to produce panel b. (TIF) [file pcbi.1012005.s012.tif]

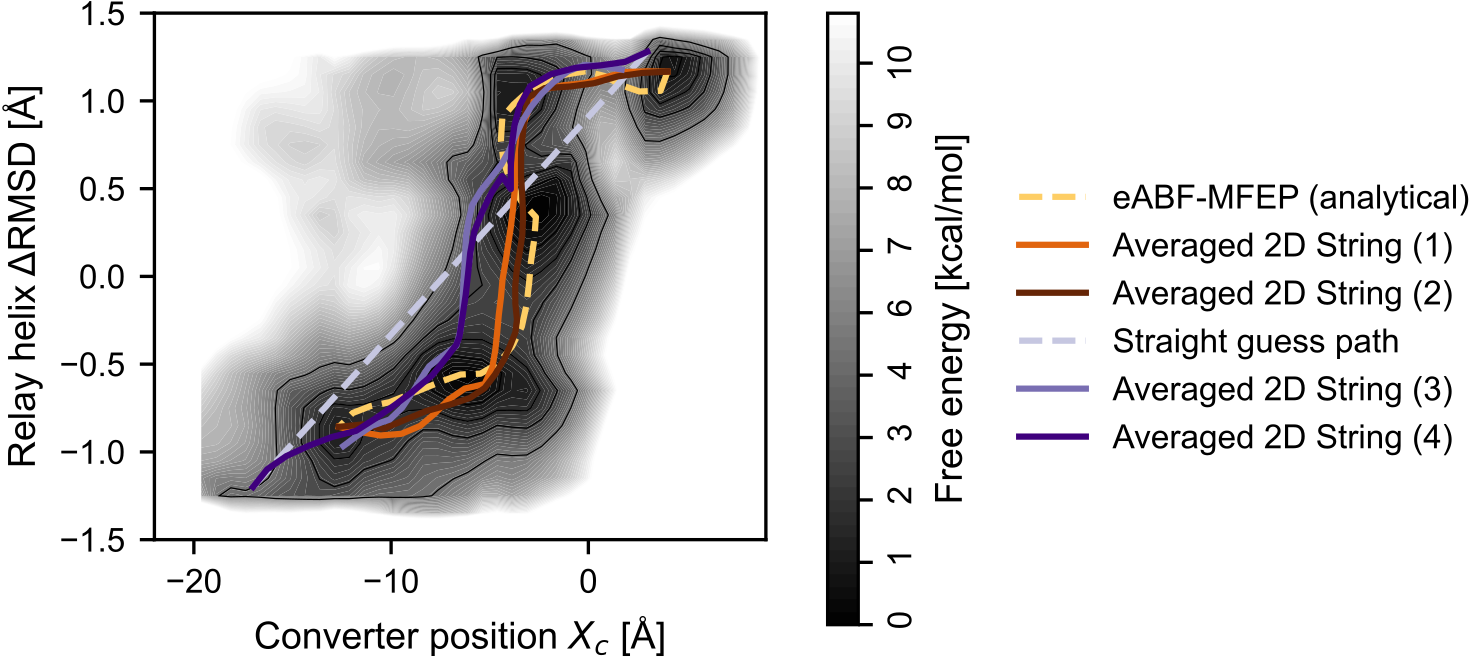

Supplement: S7 Fig — (TIF) [file pcbi.1012005.s013.tif]

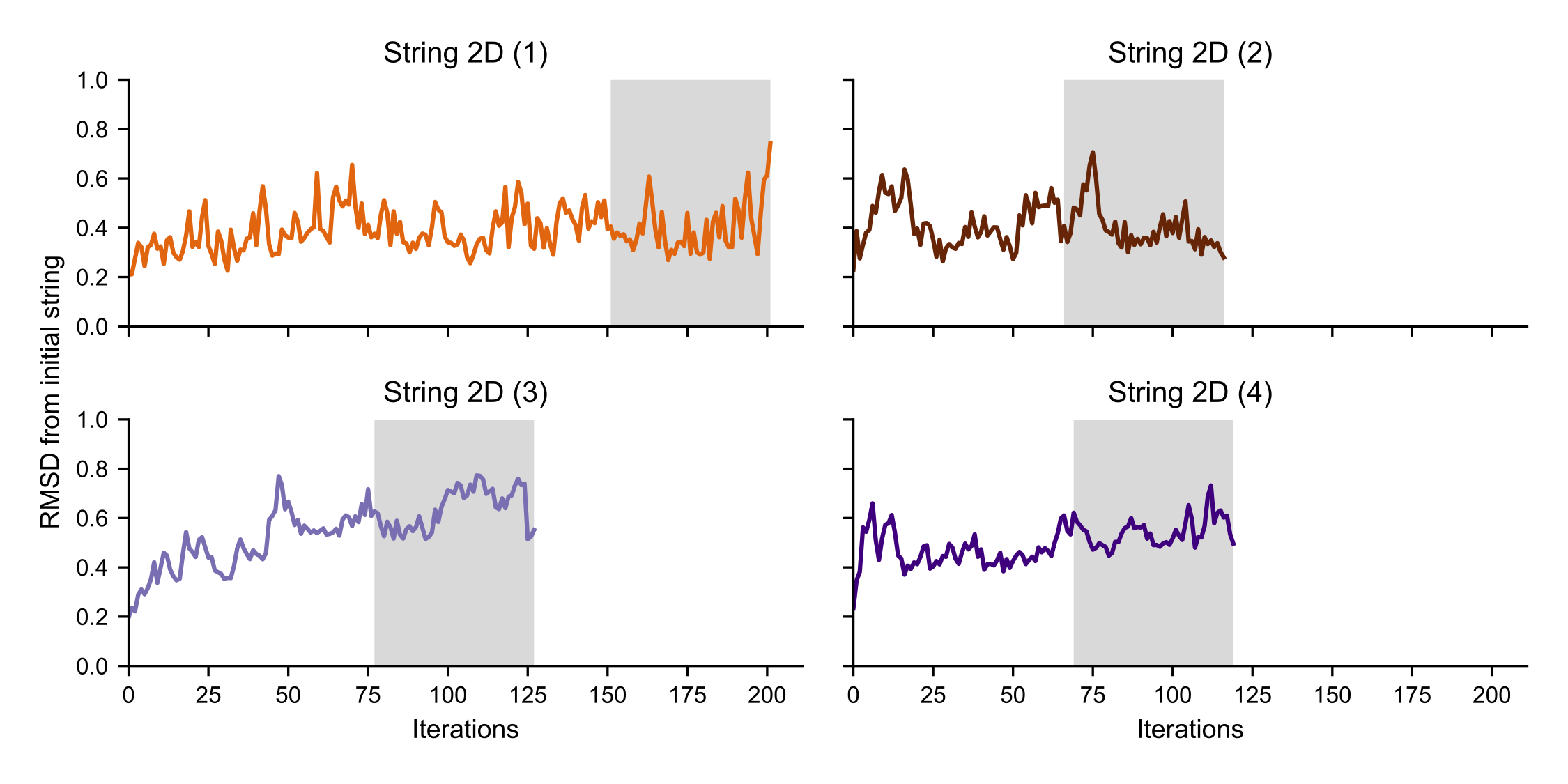

Supplement: S8 Fig — Shown is the RMSD in normalized CVs from the initial path. The greyed area shows the last 50 iterations, over which the average strings pictured in S7 Fig are computed. (TIF) [file pcbi.1012005.s014.tif]

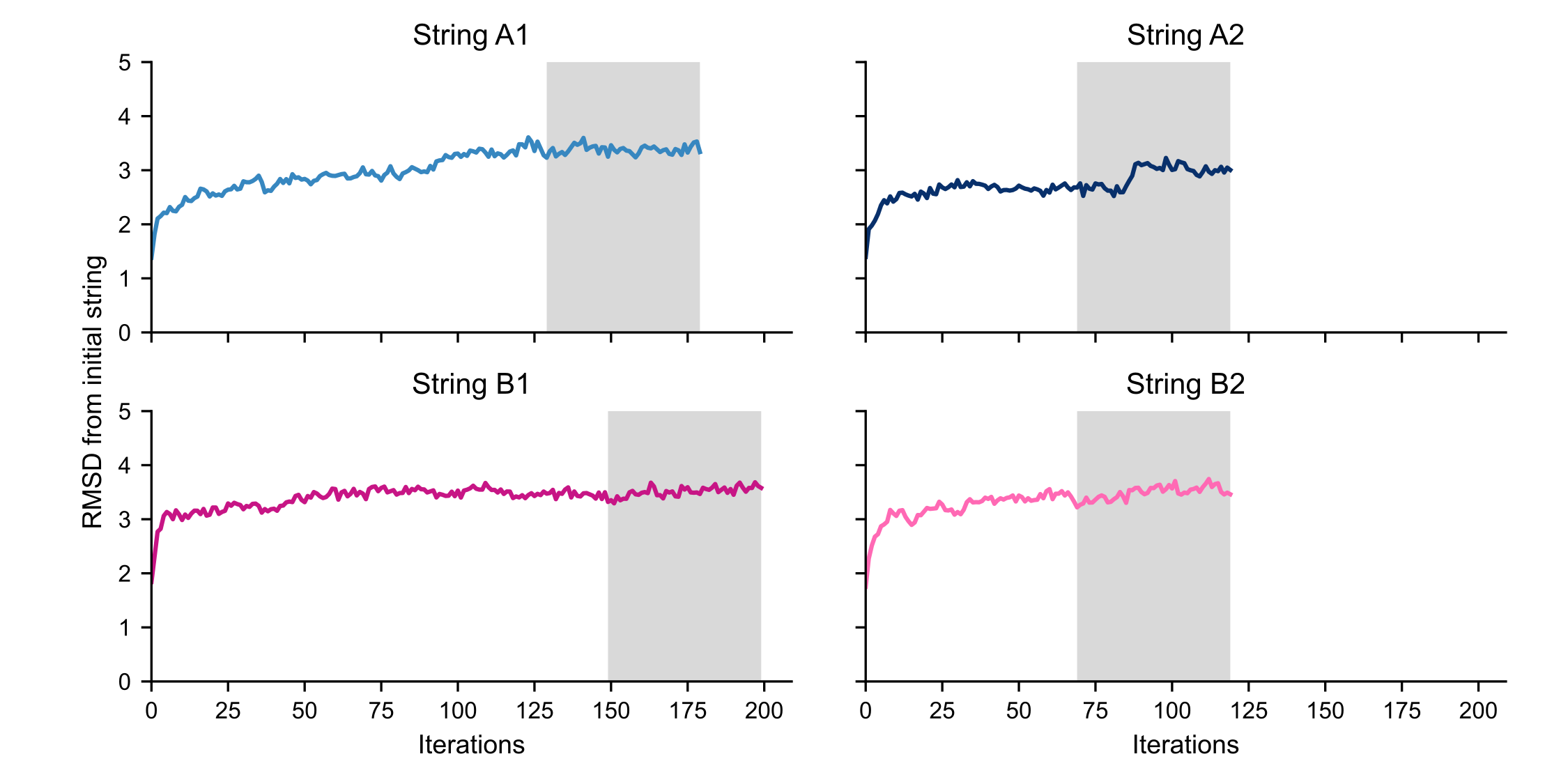

Supplement: S9 Fig — Shown is the RMSD in normalized CVs from the initial path. The greyed area shows the last 50 iterations, over which the average strings are computed. (TIF) [file pcbi.1012005.s015.tif]

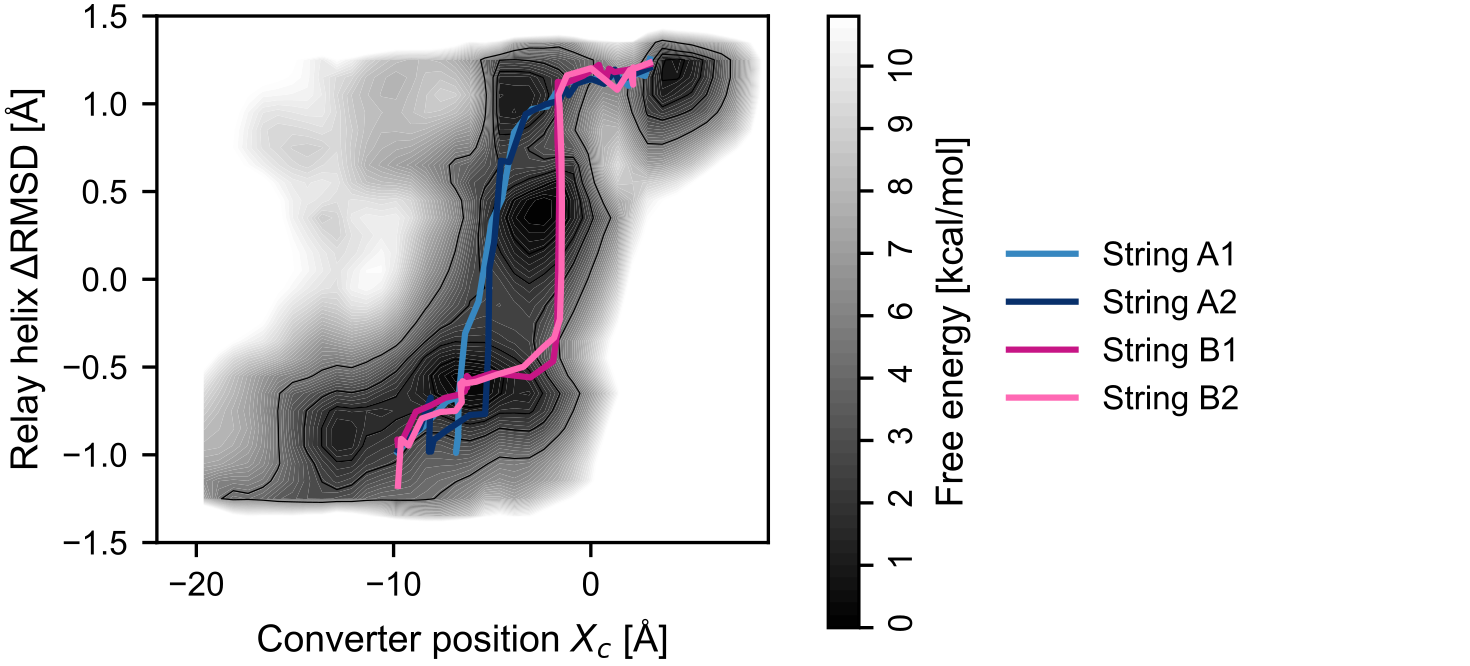

Supplement: S10 Fig — (TIF) [file pcbi.1012005.s016.tif]

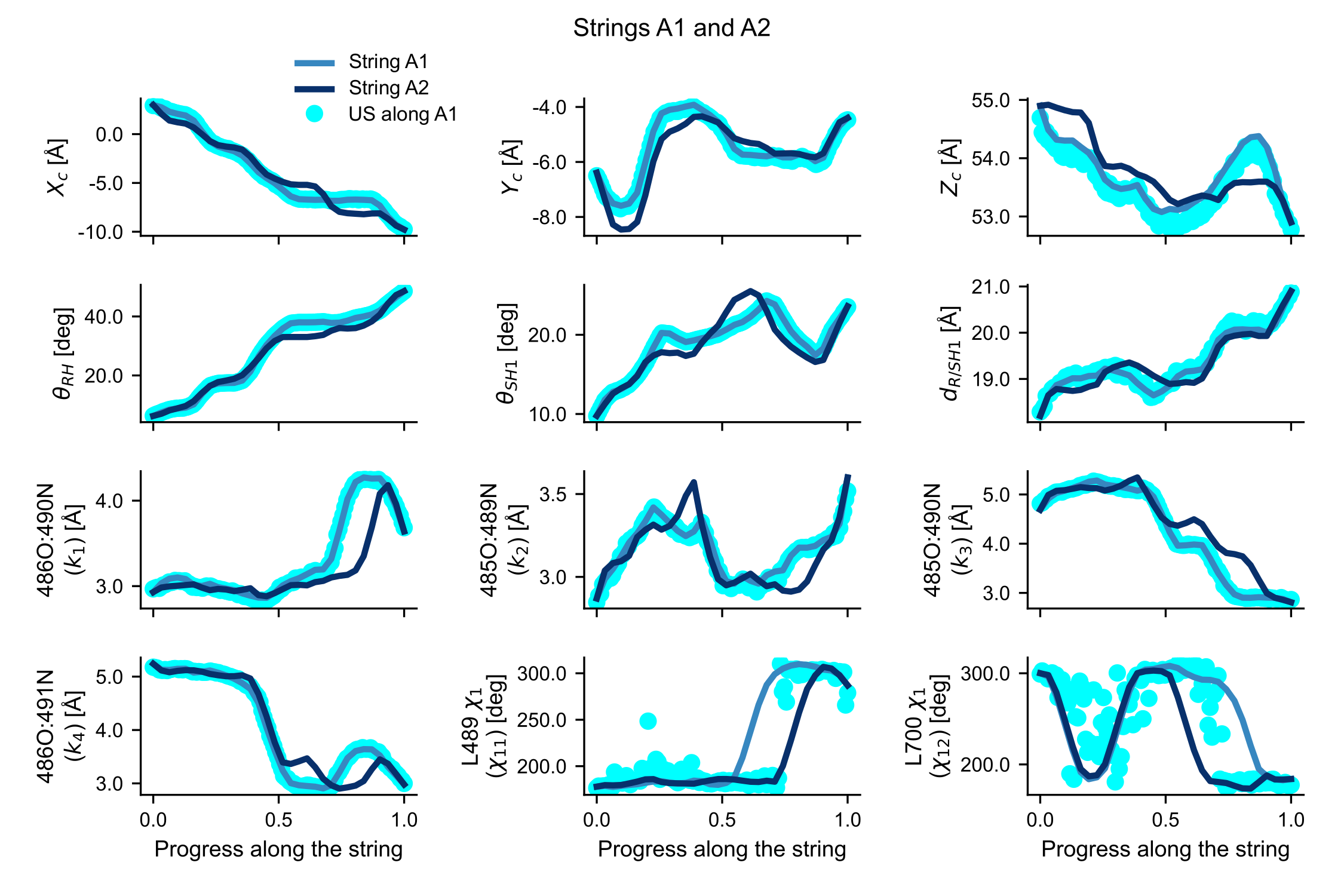

Supplement: S11 Fig — (TIF) [file pcbi.1012005.s017.tif]

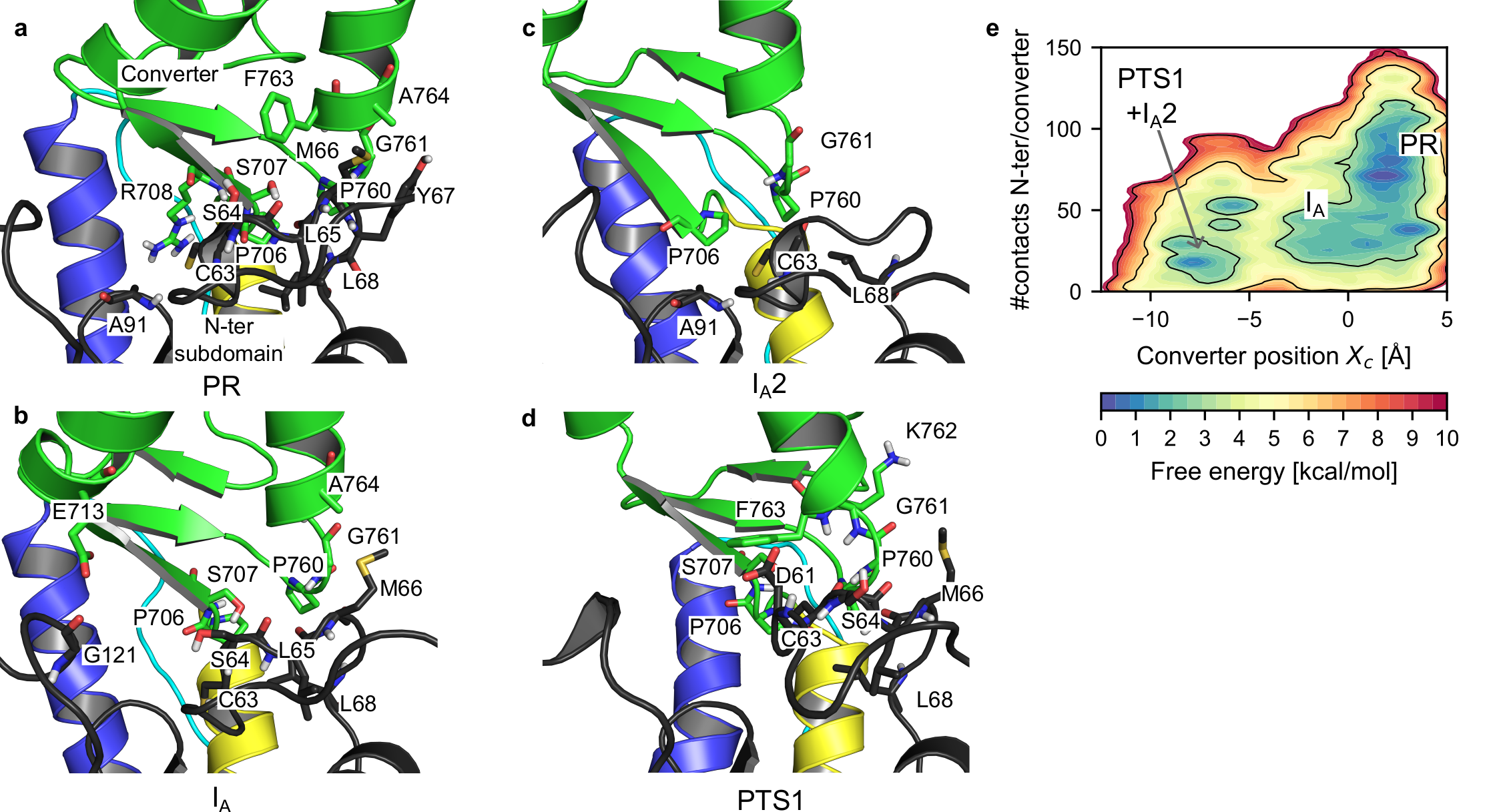

Supplement: S12 Fig — (a-d) Close-up on the converter/N-ter interface in representative frames of the metastable states sampled in umbrella sampling along String A1. Residues involved in contacts are shown as sticks. For clarity, non-polar hydrogens are not shown. (e) PMF along Xc and the number of contacts estimated with MBAR from umbrella sampling along string A1. A contact is defined as two heavy atoms being closer than 4.5 Å. Frames are the same as the ones from Fig 3. (TIF) [file pcbi.1012005.s018.tif]

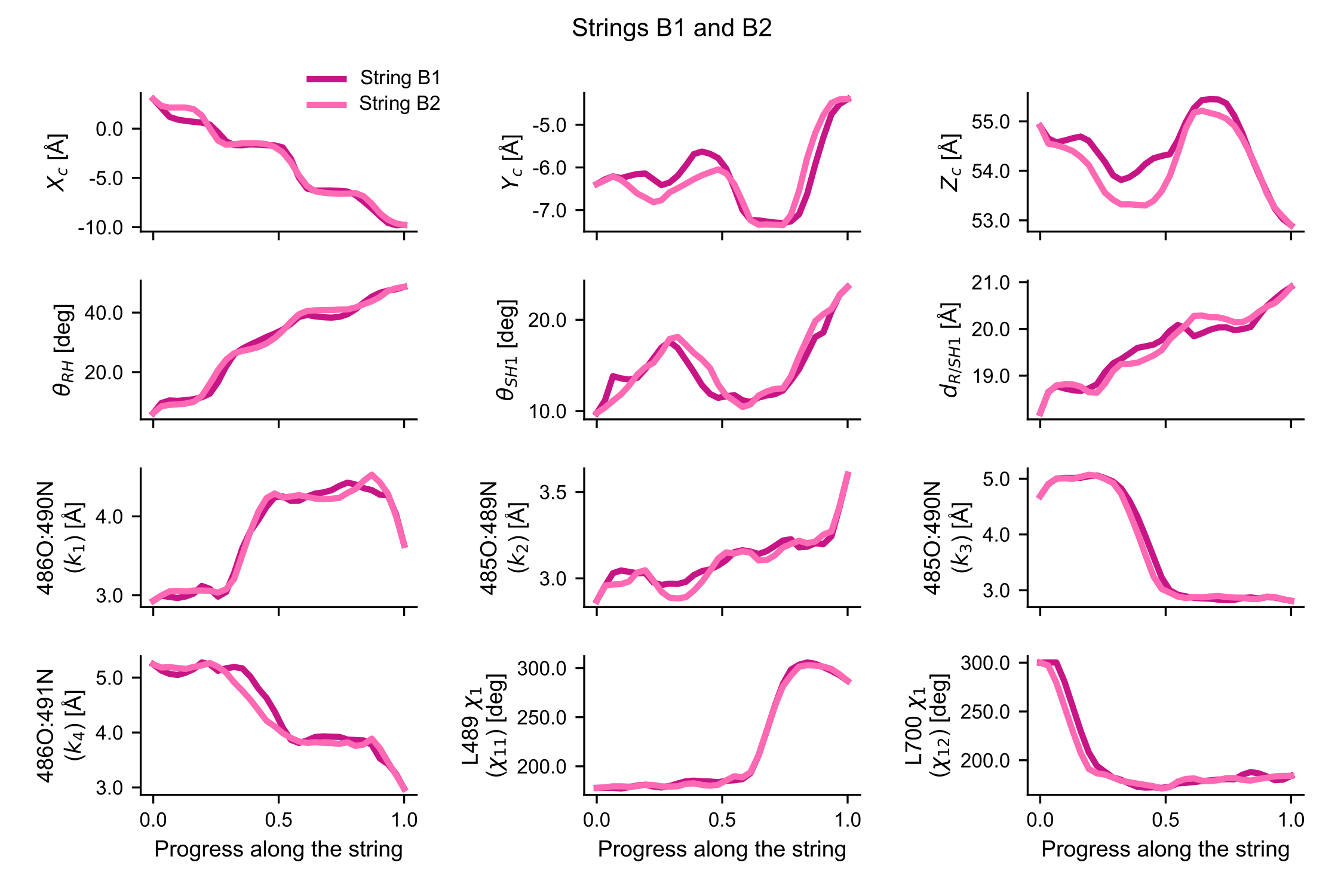

Supplement: S13 Fig — (TIF) [file pcbi.1012005.s019.tif]

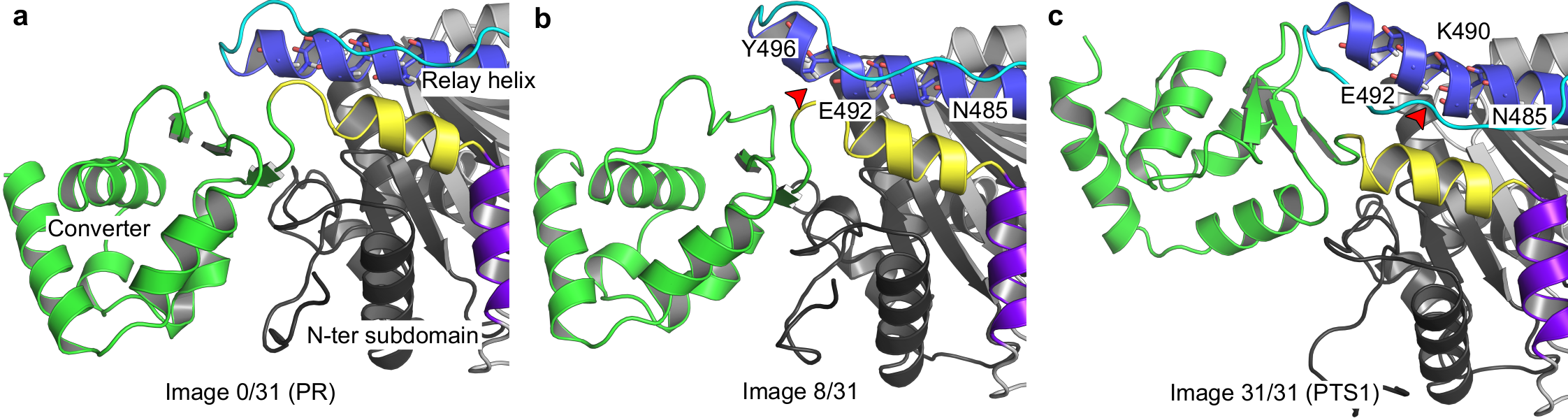

Supplement: S14 Fig — (a) Initial state (PR-like): the Relay helix is straight and intact, the converter interacts with the N-terminal subdomain. (b) α = 0.25: a secondary kink in the Relay helix (red arrow) accommodates converter movement. The converter is still in contact with the N-terminal subdomain. (c) Final state (PTS-like): the converter has moved and broken the contacts, and the canonical kink (red arrow) in the Relay helix has formed. Shown are the last simulation frames of the last iteration of string B1 optimization, for images indicated at the bottom of each panel. The complete sequence of structures along string B1 is available in Zenodo. (TIF) [file pcbi.1012005.s020.tif]

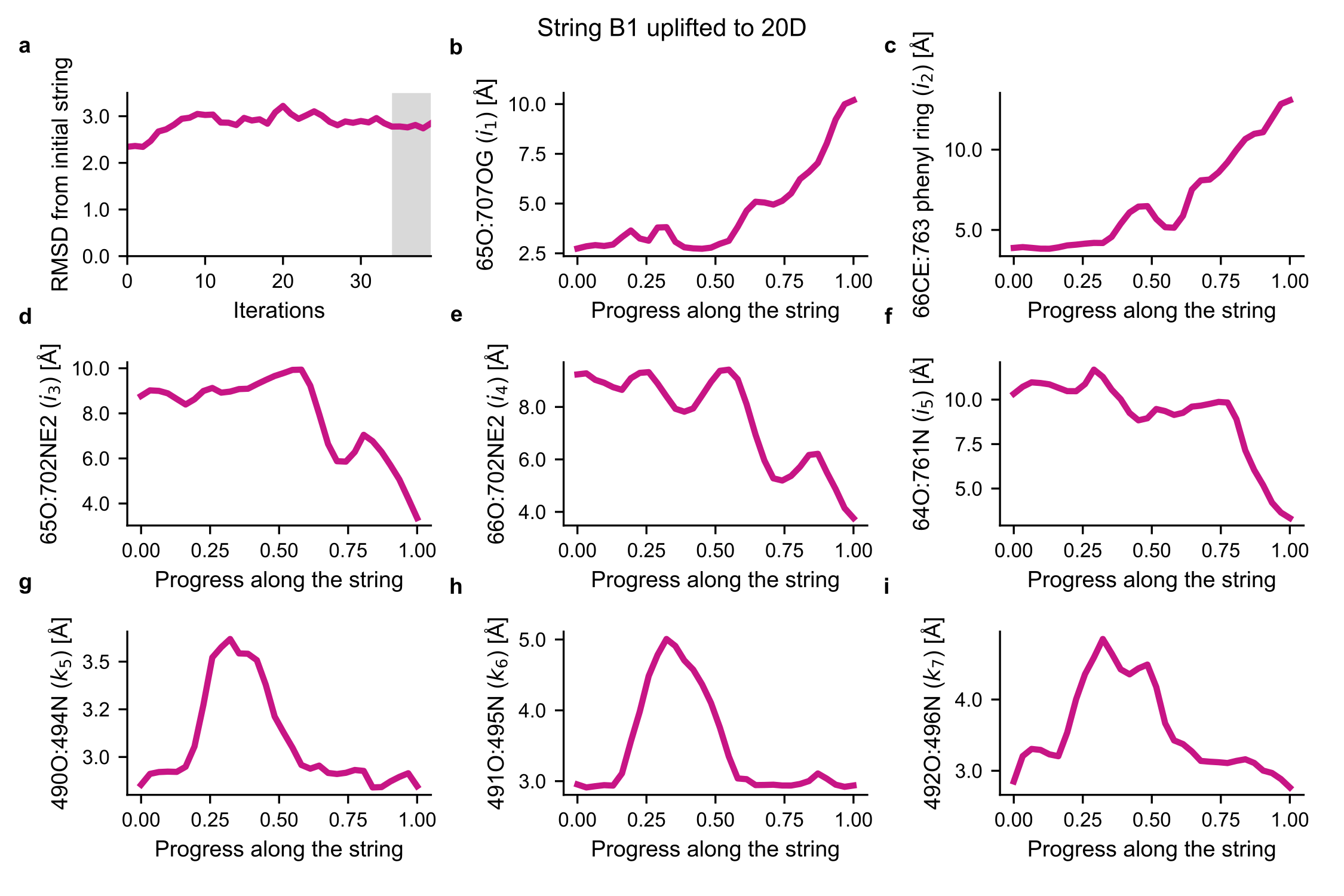

Supplement: S15 Fig — (a) Convergence of the CVSM calculation in 20D CV-space. (b-f) Evolution of converter/N-terminal distances along the converged, averaged 20D string. (g-i) Evolution of the RH backbone distances describing the secondary kink along the converged, averaged 20D string. Panels b and c show that contacts between the converter and the N-terminal subdomain are preserved until about halfway along the transition, while panels g-i show that a secondary kink forms in the RH from about 0.25 to 0.50 progress along the string. (TIF) [file pcbi.1012005.s021.tif]
